# Machine Learning Enabled Reusable Adhesion, Entangled Network-Based Hydrogel for Long-Term, High-Fidelity EEG Recording and Attention Assessment

**DOI:** 10.1007/s40820-025-01780-7

**Published:** 2025-05-29

**Authors:** Kai Zheng, Chengcheng Zheng, Lixian Zhu, Bihai Yang, Xiaokun Jin, Su Wang, Zikai Song, Jingyu Liu, Yan Xiong, Fuze Tian, Ran Cai, Bin Hu

**Affiliations:** 1 Key Lab of Brain Health Intelligent Evaluation and Intervention, Beijing Institute of Technology, Beijing, 100081 People’s Republic of China; 2 Analysis & Testing Center of Fangshan District, Beijing Institute of Technology, Beijing, 100081 People’s Republic of China; 3 School of Information Science and Engineering, Lanzhou University, Lanzhou, 730000 People’s Republic of China

**Keywords:** Entangled network, Reusable adhesion, Epidermal sensor, Machine learning, Attention assessment

## Abstract

Due to their high mechanical compliance and excellent biocompatibility, conductive hydrogels exhibit significant potential for applications in flexible electronics. However, as the demand for high sensitivity, superior mechanical properties, and strong adhesion performance continues to grow, many conventional fabrication methods remain complex and costly. Herein, we propose a simple and efficient strategy to construct an entangled network hydrogel through a liquid–metal-induced cross-linking reaction, hydrogel demonstrates outstanding properties, including exceptional stretchability (1643%), high tensile strength (366.54 kPa), toughness (350.2 kJ m^−3^), and relatively low mechanical hysteresis. The hydrogel exhibits long-term stable reusable adhesion (104 kPa), enabling conformal and stable adhesion to human skin. This capability allows it to effectively capture high-quality epidermal electrophysiological signals with high signal-to-noise ratio (25.2 dB) and low impedance (310 ohms). Furthermore, by integrating advanced machine learning algorithms, achieving an attention classification accuracy of 91.38%, which will significantly impact fields like education, healthcare, and artificial intelligence.

**Supplementary Information:**

The online version contains supplementary material available at 10.1007/s40820-025-01780-7.

## Highlights

- A dual-network hydrogel (PGEH) cross-linked via liquid metal induction was developed exhibiting remarkable mechanical properties and skin-temperature-triggered on-demand adhesion capabilities.
- The PGEH capacitive sensor demonstrates exceptional sensitivity (1.25 kPa), rapid dynamic response (30 ms), and long-term cycling stability (20,000 cycles), enabling precise monitoring of human motion and reliable signal transmission.
- Low-impedance electrophysiological sensor (310 ohms) maintains 14-day signal fidelity (25.2 dB), paired with machine learning-based attention monitoring (91.38% of accuracy) for real-time cognitive feedback in focus-demanding scenarios.

**Supplementary Information:**

The online version contains supplementary material available at 10.1007/s40820-025-01780-7.

## Graphical abstract

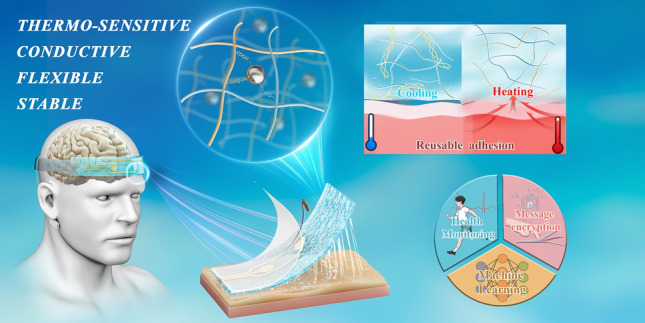

## Introduction

Hydrogel epidermal electrodes play a crucial role in the transmission of electrophysiological signals [1–3]. In systems designed for the collection and processing of physiological signals, hydrogels serve as electrode materials, offering exceptional biocompatibility and effectively minimizing the risk of allergic reactions or rejection when in contact with human skin [4–6]. These epidermal electrodes, composed of conductive hydrogels, exhibit properties akin to natural skin, such as softness, moisture retention, and deformability [7, 8]. Consequently, they have garnered significant interest in the realm of flexible epidermal electronics, with applications ranging from artificial skin to physiological monitoring [9–11]. Hydrogel electrodes are capable of accurately capturing human motion and physiological signals, such as electrocardiograms (ECG) [12], electromyograms (EMG) [13, 14], and electroencephalograms (EEG) [15]. They are extensively utilized in diagnosing cardiovascular diseases, neurological therapies, health monitoring, and human–machine interaction fields [16–18]. While traditional wet electrodes, such as Ag/AgCl gel electrodes, can reduce interface impedance to some degree, but their prolonged use often lead to signal loss and complications, including localized skin sensitization, irritation, and rashes [19, 20]. Therefore, to facilitate the long-term and accurate collection of electrophysiological signals, it is imperative to develop flexible electrodes that address these limitations.

Gelatin is a biomolecule derived from the thermal denaturation of collagen, characterized by excellent biocompatibility, temperature sensitivity (26–30 °C), water solubility, adhesion, and cost-effectiveness [21, 22]. The adhesive properties of gelatin stem from the abundance of reactive groups present in its amino acid side chains, including amino, hydroxyl, carboxyl, and sulfhydryl groups [23, 24]. Leveraging these temperature-sensitive properties, Wang [21] and Li et al. [25] reported the development of skin-coatable biogel that facilitates in situ gelation to the skin interface during EEG acquisition and can be easily removed post-use. However, the mechanical properties and stability of this biogel are not optimal. Like other single-network hydrogels [26, 27], gelatin hydrogels typically display lower strength and poor stability, which significantly constrains their application in dynamic signal monitoring.

In recent years, novel polymer network strategies such as interpenetrating networks [28], nanocomposites [29, 30], and dual-network [31] structures have been reported. These typically consist of interpenetrating brittle and ductile networks. Zhang et al. [32] reported a gelatin/polyacrylamide dual-network hydrogel in which the brittle network breaks and dissipates energy during stretching while the ductile network retains the memory of the initial state. This design enhances the hydrogel’s fatigue resistance. Additionally, Gong et al. [33] proposed a hydrogel toughening strategy that utilizes crystalline polymer entanglement, further improving the mechanical properties of hydrogels through the entanglement effect associated with dual networks. However, achieving the necessary electrical conductivity for effective electrophysiological signal acquisition remains challenging. Conductive hydrogels (such as PEDOT:PSS [34], poly(ionic liquids [35]) possess certain levels of conductivity, but they face issues like brittleness, irreversible swelling, and lack of dynamic adhesion. Their electrical performance has not yet met the requirements for effective high-fidelity electrophysiological signal collection. Furthermore, the addition of conductive materials, such as conductive nanoparticles and free ions, can significantly enhance the conductivity of hydrogels [36]. Among these, liquid metals are considered superior candidates due to their ultra-high conductivity (approximately 3.4 × 10^6^ S m^−1^) [37], self-healing properties, biocompatibility, and mechanical compliance. However, in the realm of electrophysiological signal collection, there remains a notable gap in the development of dual-network hydrogel electrodes that feature intelligent adhesion and the capability for on-demand removal, particularly following prolonged, high-fidelity collection of EEG signals. This highlights the pressing need for electrodes that can be gently removed while also ensuring reusability. Furthermore, ensuring the wearing comfort and portability of data acquisition devices while achieving high-fidelity EEG acquisition and long-term classification and monitoring of attention also pose significant challenges.

**Fig. 1:**
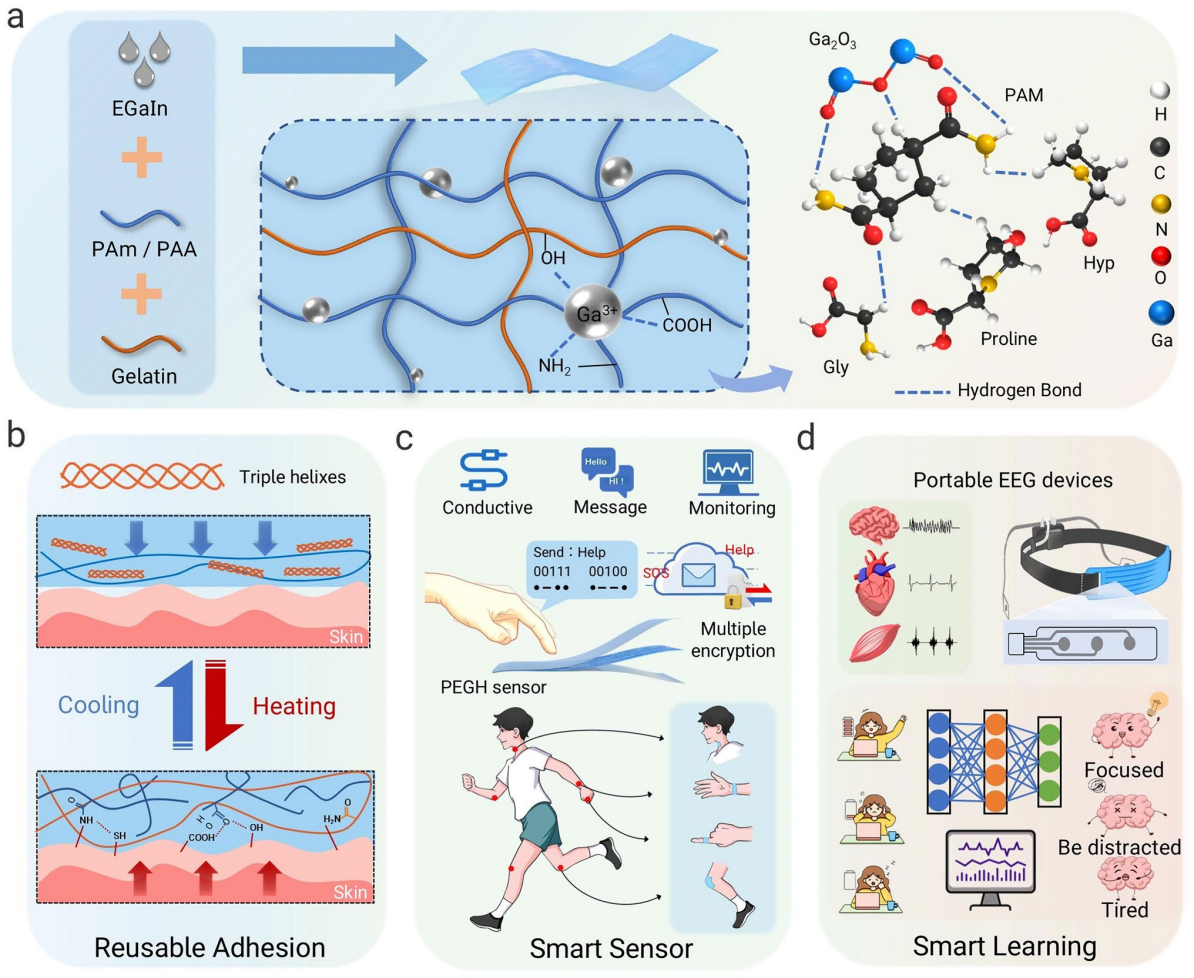
Construction and application of the PGEH. **a** Schematic of the preparation process for the PGEH, which incorporates a double cross-linked network and ball-and-stick model. **b** Illustration of the reusable adhesion mechanism used by the PGEH. **c** Schematic depicting the use of high-performance PGEH sensors in human–computer interaction. **d** Schematic of the application of the PGEH for physiological electrical signal acquisition and a portable EEG collection headband, along with attention assessment using machine learning methods

This study aims to develop a high-performance conductive hydrogel, specifically a polyacrylamide/gelatin/EGaIn hydrogel (PGEH), which is suitable for applications in artificial skin and physiological monitoring electrodes. Gelatin, as a temperature-sensitive material, offers significant adhesion properties, while an entangled gelatin polyacrylamide network enhances the overall toughness of the hydrogel. This entangled network structure effectively addresses the issue of softening and breaking gelatin molecular chains in high-temperature environments, giving them stable intelligent adhesion properties. Furthermore, the activation and cross-linking of liquid metal nanoparticles within PGEH confer properties that eliminate the need for additional cross-linking agents (Fig. 1, upper image). Due to its innovative design, PGEH demonstrates intelligent adhesion characteristics, low interfacial resistance, and high sensitivity. It exhibits long-term stable adhesion capabilities, with a strength of 104 kPa, allowing for 30 cycles of stable and repeated adhesion, which enables it to conformally and securely adhere to human skin (Table S2 and Fig. 1, first lower image). As an electronic skin, PGEH also exhibits excellent sensing properties and is capable of transmitting encrypted information, such as Morse code and binary code (Table S3 and Fig. 1, second lower image). Additionally, as a physiological monitoring sensor, it effectively captures high-quality epidermal electrophysiological signals, achieving a high signal-to-noise ratio (SNR) of 25.2 dB and low impedance of 310 ohms (Table S4). To improve signal quality, we applied a 500th-order FIR low-pass filter (0.5–45 Hz) to remove baseline wander and high-frequency interference, and used a DWT-Kalman model to eliminate ocular artifacts (OAs) [38, 39]. Moreover, utilizing the advanced machine learning algorithm EEGNet, we achieve an accuracy of up to 91.38% in attention assessment (Fig. 1, third lower image), facilitating personalized work suggestions and potential danger alerts for users. Our work indicates significant potential for applications in personal health monitoring, encrypted message transmission, and machine learning-assisted personalized concentration assessment systems, advancing the development of flexible smart wearable sensors.

## Experimental Section

### Materials

Type A gelatin, acrylamide (AM), and acrylic acid (AA) were sourced from Sigma-Aldrich, USA. Polyvinyl pyrrolidone (PVP, Mn = 4000) and ammonium persulfate (APS) were purchased from Macklin, China. EGaIn (composed of 75% Ga and 25% In) was provided by Shenyang Jiabei Commercial Technology Co., Ltd. (China). Silver ink was purchased from Shanghai Mifang Electronic Technology Co., Ltd. (China). Silicone rubber (Ecoflex 00–30) was acquired from Smooth-On. All reagents were used as received. Deionized (DI) water was used throughout the experiment.

### Fabrication of the PGEH

First, PVP (0.1 g) and gelatin (3 g) were dissolved in 5 mL of DI water and stirred at 90 °C for 2 h. EGaIn (0.6 g) was then added to this solution, followed by sonication in an ice-water bath. Sonication was performed using a probe sonicator (1000Y, Shanghai Hannuo Instrument Co., Ltd.) at 30% amplitude, alternating 3 s on and 3 s off, for a total of 120 s to prepare conductive ink.

To prepare the PGEH, AM (5 g) and AA (0.5 g) were dissolving in DI water (5 g) and adding APS (0.016 g, 0.3 wt% per monomer) as the initiator. The mixture was then poured into a vial and stirred at 25 °C for 3 h. Next, the conductive ink was added to the solution and polymerized in an oven at 70 °C for 30 min, followed by aging at 25 °C for 6 h to complete gelation. To measure the electrical conductivity, samples of PGEH-0LM, PGEH-0.2LM (0.2 g/10 mL for EGaIn/DI water), PGEH-0.4LM (0.4 g/10 mL), PGEH-0.6LM (0.6 g/10 mL), and PGEH-0.8LM (0.8 g/10 mL) were prepared.

### Construction of the PGEH Capacitive Sensor

The PGEH capacitive sensor was designed with a sandwiched structure comprising hydrogel layers on the top and bottom and encapsulating Ecoflex within the hydrogel. The detailed structure and dimensions are shown in Fig. 4a later. Ecoflex 00–30 silicone rubber (1 g) was cast into a prefabricated 1 × 1 cm^2^ rectangular model, vacuumed to remove air bubbles, and left at room temperature for 3 h to form a layer. Conductive copper foils served as external electrodes, with the entire assembly encapsulated in VHB tape for structural integrity. The prepared PGEH of fixed dimensions (1 × 1 × 0.1 cm^3^) was then placed atop and beneath the Ecoflex silicone rubber. Conductive copper foil was attached as an external electrode. The capacitance value was measured using an LCR meter (TH2830 Shanghai Tonghui).

### Design of the PGEH Electrophysiological Signal Electrodes

Waterborne polyurethane (WPU) films of thickness 100 μm were used as substrates for the silver ink electrodes. The WPU substrates underwent ultrasonic cleaning in ethanol and were subsequently air-dried. The silver ink was inkjet-printed onto the flexible WPU film using a Microelectronic Printer (MP1100, Mifang, China) and then dried in an oven at 80 °C for 24 h. Finally, the PGEH was solidified on the surface of the printed electrode through a drop coating process. The distance between each electrode was maintained at 20 mm, with a contact area of approximately 50 mm^2^ per electrode on the skin.

### PGEH-Based Capacitive Sensor System for Cryptographic Code Transmission

The cryptographic code transmission system integrates an Arduino microcontroller, a PGEH capacitive sensor, and a lithium-polymer battery. To optimize signal integrity, shielded Dupont cables (1.27 mm^2^ cross section) were implemented to mitigate radiofrequency and electromagnetic interference during data acquisition. The operational workflow involves sequential finger presses to generate structured Morse code or binary signals, where temporal variations in contact duration (short/long presses) correspond to dots/dashes in Morse code, and press variations in contact strength (weak/strong presses) correspond to 0/1 in binary. These analog signals are digitized by the microcontroller and visualized in real time via a MATLAB GUI, providing immediate user feedback.

## Results and Discussion

### Structural and Mechanical Characterization of the PGEH

**Fig. 2:**
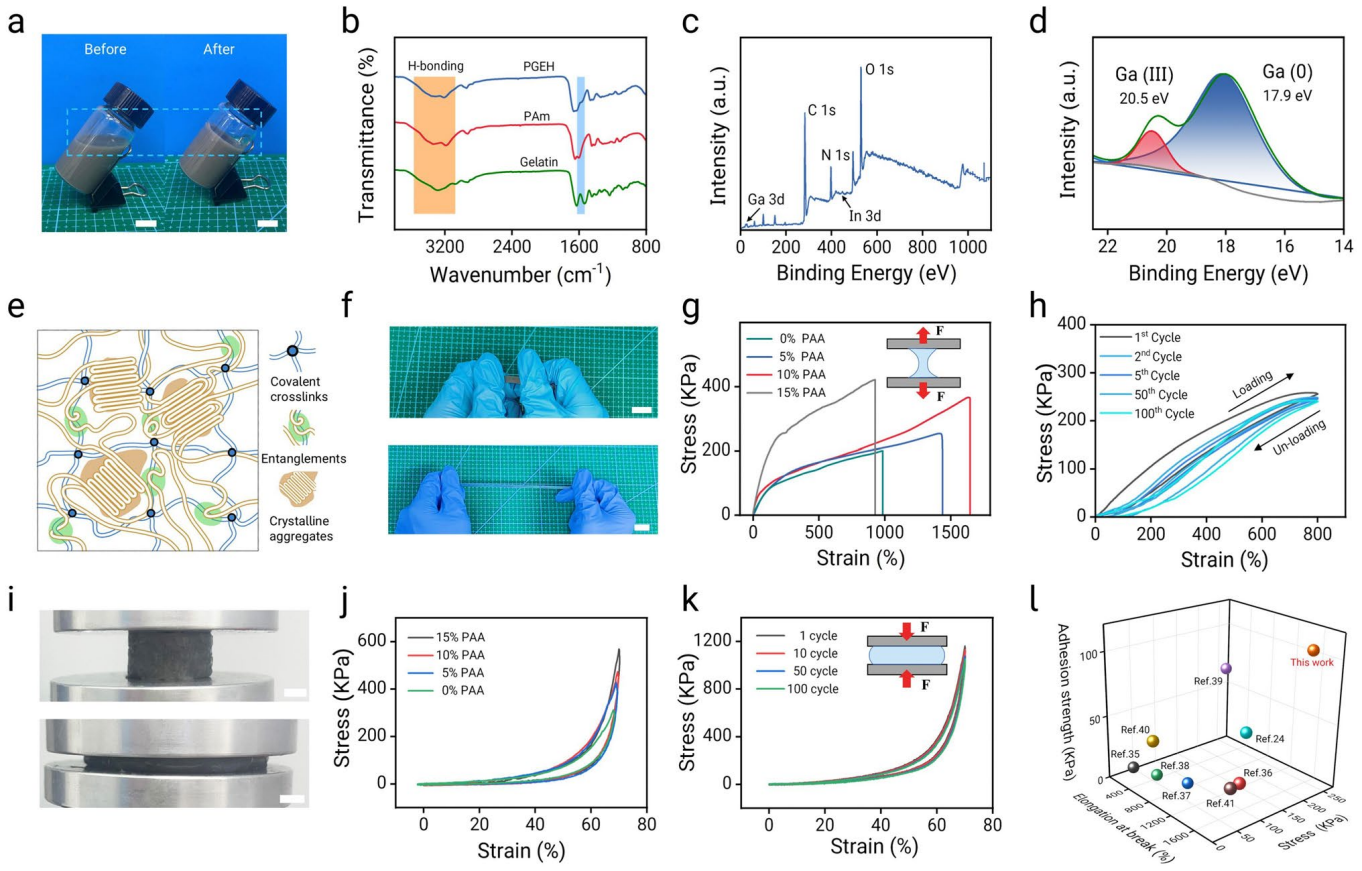
Structural and mechanical characterization of the PGEH. **a** Images of the PGEH before and after cross-linking. Scale bar: 0.5 cm.** b** Fourier transform infrared (FTIR) spectrum of the PGEH. **c**, **d** X-ray photoelectron spectroscopy (XPS) energy spectrum and high-resolution Ga 3*d* spectrum of the PGEH. **e** Schematic of the double-network entangled hydrogel structure. **f** Images of the PGEH stretching. Scale bar: 1 cm. **g** Stress–strain curve of the PGEH with varying PAA content. **h** Cyclic tensile curve of the PGEH, tensile length of 800%. **i** Images of the PGEH under compression. Scale bar: 5 mm. **j**, **k** Compression and cyclic compression curves of the PGEH with different PAA content, the compression ratio being 70%. **l** Literature comparison of the tensile strength, breaking elongation, and adhesion strength of hydrogels [25, 32, 49–54]

The ultrasonic-treated liquid metal nanoparticle conductive network was integrated into a polymer hydrogel network comprising of gelatin and PAM/acrylic acid to construct the PGEH [40]. Figure 2a shows the hydrogel both before and after cross-linking. Notably, the PGEH does not require a cross-linking agent, as it initiates the polymerization reaction of vinyl monomers through liquid metal induction [41, 42]. Figure S1a shows the three-dimensional interconnected porous structure of the hydrogel, accompanied by the dispersion of liquid metal particles within the hydrogel network. Notably, the incorporation of liquid metal-based cross-linkers induces structural densification of the hydrogel matrix [43, 44], as evidenced in Fig. S1b, c. Energy-dispersive X-ray (EDX) spectroscopic mapping (Fig. S2) reveals uniform distribution of C, O, N, Ga, and In elements in the PGEH. As shown in Figs. 2b and S3, the Fourier transform infrared spectrum reveals a distinct broad peak at 3300 cm^−1^, attributed to the absorption of water, the stretching vibration of the –OH group, and the hydrogen bonding force of Ga_2_O_3_ within the liquid metal oxide layer [45]. The absorption band located at 1650 cm^−1^ corresponds to the stretching vibration of C=O in both the amide and carboxyl groups [46]. The peaks at 1547 and 1460 cm^−1^ can be attributed to the fundamental vibrations of gelatin, displaying a similar trend in the prepared PGEH, clearly demonstrating the successful synthesis of the PGEH. X-ray photoelectron spectroscopy (XPS) of the PGEH confirmed the presence of Ga, In, and other elements (Fig. 2c). The two characteristic peaks of Ga 3*d*_3/2_ and Ga 3*d*_5/2_ correspond to the initial Ga (I) binding energy of 17.9 eV and the Ga_2_O_3_ (III) binding energy of 20.5 eV. Figure 2d illustrates that the liquid metal particles predominantly exist in the Ga_2_O_3_ state, further substantiating the hydrogen bonding interactions between the liquid metal particles and the hydrogel [47]. The spectra of other elements are shown in Fig. S4.

The PGEH exhibited a dual-network structure, comprising a physically cross-linked gelatin network as the first component and a covalently cross-linked PAM network as the second. At lower temperatures, the gelatin chains self-assemble into triple helices that entangle around the acrylamide molecular chains, thereby forming an entangled network [31, 33]. As shown in Fig. 2e, the intertwined molecular chains enhance the resilience of the hydrogel, preventing brittle fractures. By contrast, pure gelatin hydrogels, as shown in Figs. 2f and S5, typically exhibit poor tensile properties [21, 48]. The PGEH showcases extraordinary flexibility, as illustrated in Figs. 2g, S6, and S7. The incorporation of increased PAA(Polyacrylic acid) content introduces additional cross-linking sites to the PGEH. With 10% PAA, the PGEH exhibits a substantial strain capacity of 1645%, a tensile strength of 366.54 kPa, and notable toughness of 350.2 kJ m^−3^. This enhancement dramatically improves the elongation and compressive strength of the PGEH.

Figure 2h shows that the mechanical strength of the PGEH remains largely unchanged after 1, 2, 5, 50, and 100 cycles of cyclic stretching, indicating excellent durability and fatigue resistance. Moreover, the compression tests revealed that the PGEH exhibited commendable elasticity and durability across different compression ratios (Figs. 2i and S8). As shown in Fig. 2j, k, the material exhibits outstanding fatigue resistance and consistent energy dissipation during cyclic deformation, with no major hysteresis loop evident observed over a 100-cycle compression test. Compared to previously reported highly adhesive hydrogels (Fig. 2l), the PGEH concurrently exhibited high adhesive strength and exceptional mechanical properties, which are advantageous for constructing conformal contact interfaces and ensuring stable, high-quality electrical signal transmission (Table S2).

### Reusable Adhesion Properties of the PGEH.

**Fig. 3:**
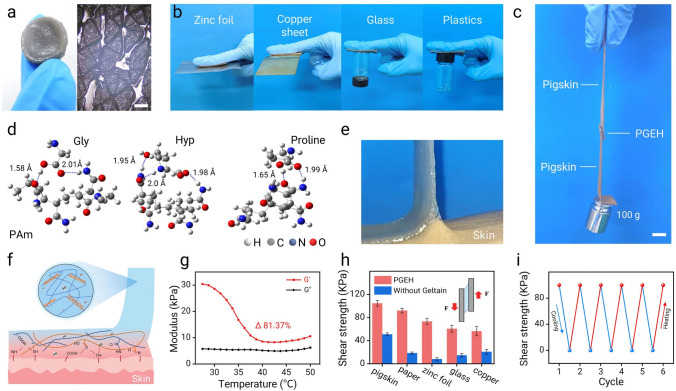
Reusable adhesion properties of the PGEH. **a** Images showing the skin texture after the removal of the PGEH (left: thumb texture, right: wrist joint texture). Scale bar: 200 μm. **b** Images of the PGEH adhesion to various substrates. **c** PGEH adhered to pig skin, demonstrating its ability to support a weight of 100 g. Scale bar: 5 mm. **d** Density functional theory (DFT) calculations illustrating the interactions between the PAM and amino acid components of gelatin. **e** Schematic of the PGEH peeling process from the skin. **f** Schematic of the internal system of the PGEH, highlighting its reusable adhesion properties influenced by temperature. **g** Comparison of the adhesion strength across different substrates. **h** Rheological test curve of the PGEH, with a scanning temperature range of 25–50 °C. **i** Adhesive strengths of the PGEH upon heating the hydrogel to 40 °C or cooling to 10 °C

The PGEH not only demonstrated exceptional mechanical properties but also exhibited significant reusable adhesion. As shown in Figs. 3a and S9, the PGEH hydrogel closely and stably adhered to the wrinkled tissue surfaces, effectively replicating the skin texture after in situ cooling. Moreover, as shown in Fig. 3b, c, the PGEH hydrogel displayed remarkable adhesion properties across a variety of substrates—including aluminum foil, copper sheets, and glass, as well as excellent adhesion to human skin (Fig. 3e). This tissue-like compliance is crucial for electrodes, as it not only addresses the biomechanical mismatch between the skin and the electrode interface but also minimizes the impact of motion on the electrode performance.

Using density functional theory (DFT) calculations, it was determined that a considerable interaction force exists between the PAM and gelatin chains (Figs. 3d and S10, Table S1) [55]. These interactions primarily involved hydrogen bonding and electrostatic forces between the PAM backbone and the carbonyl/hydroxyl groups of gelatin. The resulting entangled network ensured uniform dispersion of gelatin’s triple-helical chains within the hydrogel, facilitating rapid thermal responsiveness. Crucially, this adhesion mechanism (Fig. 3f) arises directly from the dynamic nature of these interactions. When the PGEH contacts warm skin (30–40 °C), body heat triggers partial decomplexation of gelatin. This releases mobile gelatin chains that expose functional groups (e.g., -OH, -NH_2_), enabling strong interfacial bonds with the skin surface. The adhesion strength was quantified across different substrates (Fig. 3h), with values of 104.7 kPa (pig skin), 92.0 kPa (glass), and 56.4–73.3 kPa (metals), demonstrating its versatility. Notably, adhesion remained stable over 30 cycles (Fig. S11), confirming its robustness.

The thermal sensitivity of this adhesion mechanism is further validated by rheology (Fig. 3g). As the temperature exceeded 30 °C, the storage modulus (G′) of PGEH dropped sharply (81.37%), confirming that the PGEH exhibited optimal performance within the temperature range of 30–40 °C, highlighting its sensitivity to heat [56]. In contrast, by both the G′ and loss modulus (G″) of the gelatin hydrogel displayed an upward trend without a pronounced temperature-dependent response, as shown in Fig. S12. Moreover, it should be noted that the G′ of the PGEH consistently exceeded the loss modulus throughout the temperature scan. Consequently, even if the gelatin molecules partially melted, the hydrogel retained its solid elasticity as long as the temperature remained below 50 °C [55]. Upon contact with warm skin, the body temperature initiated the decomplexation of gelatin, allowing the freely movable gelatin chains within the hydrogel to expose numerous functional groups that form multiple interfacial bonds with the skin surface, resulting in strong adhesion (Fig. 3f).

Additionally, the PGEH demonstrated stable cyclic adhesion and shedding properties under temperature stimulations, fulfilling the requirements for repeated use in daily life [21, 48]. Repeated adhesion tests conducted on pig skin at varying temperatures revealed that the hydrogel exhibited a high average adhesion strength of 101.3 kPa at a body temperature of 37 °C. However, at 10 °C, its adhesion force was considerably reduced (Fig. 3i). This finding further substantiates that painless peeling of PGEH can be effectively achieved through cooling treatments, including cold-water rinsing or ice application (Figs. S13 and S14, Movie S1), while maintaining excellent flexibility under low-temperature conditions, mitigating the risk of secondary damage to new tissue typically caused by traditional adhesive removal methods. Notably, the skin adhesion capacity of the PGEH surpassed that of previously reported temperature-sensitive hydrogel adhesives (Table S2). These research findings confirmed that the PGEH could serve as a mild hydrogel adhesive dressing offering high adhesion and easy removal. Moreover, the PGEH could play a major role in the medical field in the future, suitable for wearable monitoring devices and medical equipment accessories.

### Multifunctional Construction of PGEH Sensors

**Fig. 4:**
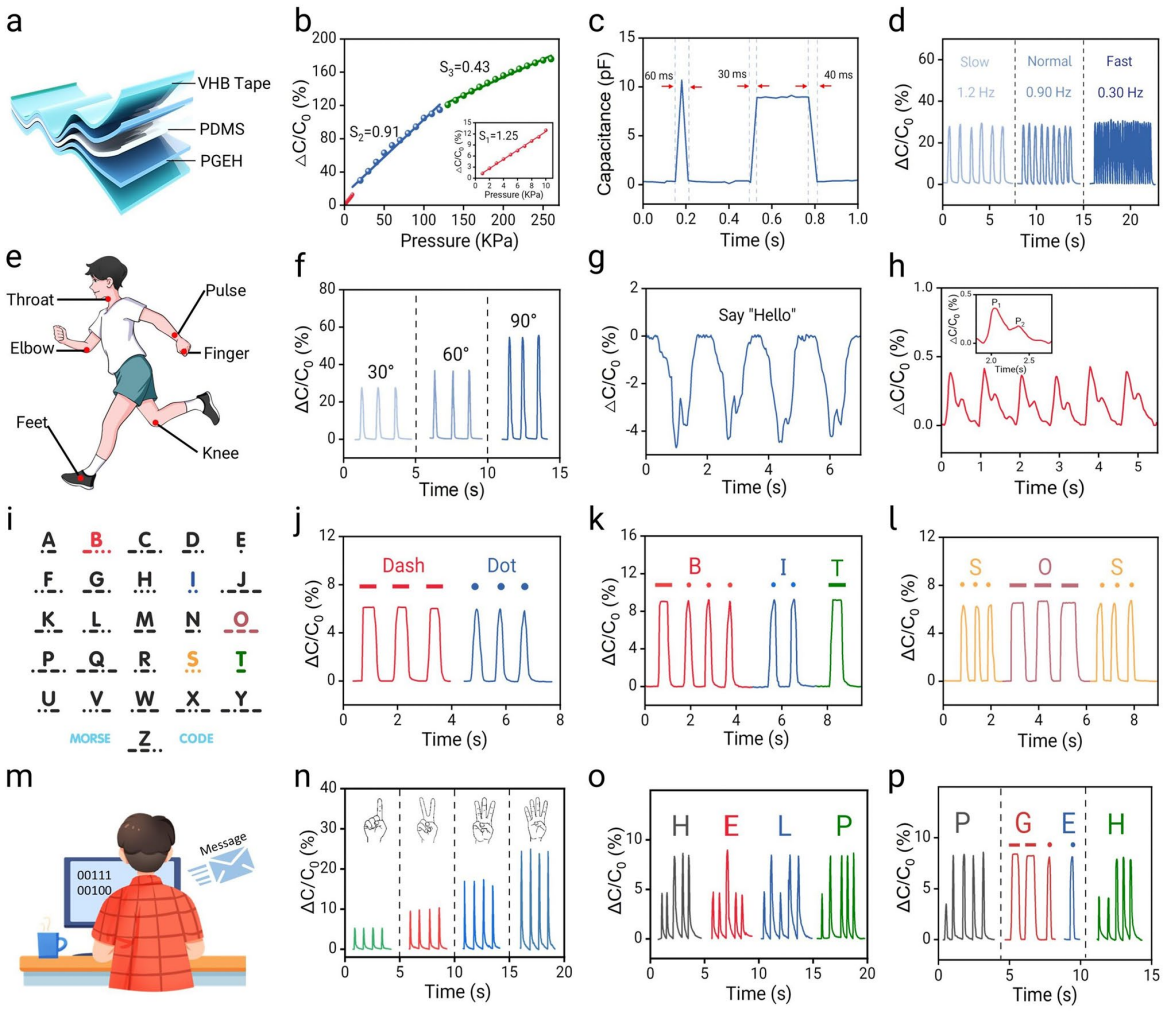
Multifunctional construction of PGEH sensors. **a** Schematic of the PGEH epidermal sensor assembly. **b** Pressure sensitivity of the PGEH sensor, covering a pressure range of 0–260 kPa. **c** Response time of the PGEH sensor. **d, f** Relative capacitance changes of the PGEH sensor fixed on the finger joint at various frequencies and bending angles. **e** Schematic showing human motion detection. **g** Relative capacitance changes of the PGEH sensor affixed to the Adam’s apple during vocal cord vibrations. **h** PGEH sensor positioned on the radial artery for pulse wave monitoring without external pressure. **i** Schematic of the Morse code alphabet. **j** Demonstration of basic Morse code symbols. **k** Transmission of the word "BIT" and **l** "SOS" as encrypted messages using Morse code. **m** Diagram of sending binary information. **n** Relative capacitance changes of the PGEH sensor in response to different numbers of fingers. **o** Transmission of the encrypted message "HELP" using binary code principles. **p** Hybrid encrypted message transmission using the PGEH sensor

The PGEH exhibits excellent temperature-triggered adhesion and separation properties, making it an ideal candidate for use as a biological interface material, facilitating a seamless fit between epidermal bioelectronic devices and the skin surface. To validate this functionality, as shown in Fig. 4a, a conductive hydrogel was synthesized by integrating liquid metal into the PGEH, and a flexible capacitive sensor was constructed using polydimethylsiloxane (PDMS) as the dielectric layer for comprehensive monitoring of human activities [57]. The capacitance could be expressed as C = εA/d, where ε denotes the dielectric constant, d denotes the distance between two parallel electrodes, and A denotes the contact area between the dielectric layer and electrode [58]. Figure 4b illustrates the relative capacitance change of the PGEH-based epidermal sensor across a broad pressure range (260 kPa), achieving a sensitivity of up to 1.25 and a response time as rapid as 30 ms (Fig. 4c). These performance metrics are commendable when compared to many previously reported high-performance sensors (Table S3).

First, the PGEH sensors were employed to detect significant human movements. Figure 4d, f shows that the sensor could be installed on the human finger to measure both the bending angle and speed of finger movements. The inductive response increased with the bending angle, whereas the frequency increased with speed, indicating that the sensor could accurately distinguish between different bending angles and speeds. Similarly, as shown in Fig. S15, these sensors were affixed to the wrist and knee to monitor bending movements. The clear and repeatable signals confirmed the sensor’s reliable detection abilities. Moreover, the performance remained stable for 20,000 cycles with the capacitance deviation value being just 1.2% (Fig. S16). Consequently, the sensor was capable of monitoring injured joints across various bending angles and could provide essential diagnostic information for postoperative rehabilitation training. Moreover, the sensor was also capable of detecting subtle human movements, as shown in Figs. 4g and S17, showcasing the real-time sensing performance of the PGEH sensor in detecting throat vibrations. The clear and reproducible signals validated the sensor’s reliability, highlighting its potential application in the field of speech recognition. Moreover, the performance of the human wrist pulse is considered an important parameter that offers critical diagnostic information regarding heart rate and arterial health, underscoring the need for timely monitoring. As shown in Fig. 4h, the PGEH sensor was positioned on the wrist artery to facilitate real-time monitoring of the pulse, yielding a normal and regular pulse waveform with a frequency of approximately 80 beats per minute (bpm). The sensor’s high sensitivity allowed for the capturing of detailed pulse waveforms, including the systolic (P_1_) and diastolic (P_2_) waves. The radial augmentation index (AIr), defined as the ratio of P_2_ to P_1_, is critical for diagnosing vascular aging and arteriosclerosis [49]. In this study, the calculated ratio from the measured waveforms was 0.48, consistent with normal values for healthy adult males.

PGEH sensors can also be employed to transmit encrypted information. Morse code uses a combination of dots and dashes to represent the letters of the English alphabet (Fig. 4i) [59]. In this encoding system, as shown in Fig. 4j, short presses generating spikes and sustained presses producing straight lines represent dots and dashes, respectively. Words such as "BIT" and "SOS" can be conveyed through corresponding electrical signals (Fig. 4k, l). Additionally, the PGEH sensor facilitates encrypted information transmission based on the principles of binary code. Changes in capacitance signals, resulting from different numbers of fingers (Fig. 4m, n), can be detected by high-sensitivity PGEH pressure sensors, with the range of capacitance changes depicted in Fig. S18. As shown in Fig. 4o, the message "HELP" was achieved through alternating finger presses using one and two fingers on the high-sensitivity PGEH sensor. Moreover, as shown in Fig. 4p, by integrating the two encryption methods, the PGEH sensor could be operated with one hand to transmit more complex encrypted information (Fig. S19 and Movies S2-S4), thereby securing confidential data. This innovation could offer customized solutions for encrypted information transmission and broaden the application scope of flexible wearable sensors.

### Application of PGEH-based Epidermal Sensors

**Fig. 5:**
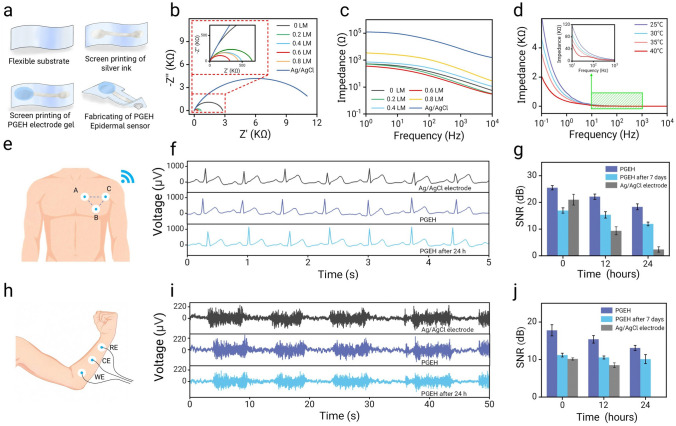
Application of PGEH-based epidermal sensors. **a** Fabrication process of the PGEH electrodes. **b** Nyquist plot of the PGEH with varying EGaIn content. **c** Bode plot of the PGEH with different liquid metal content. **d** Bode plots of the PGEH at temperatures ranging from 25–40 °C. **e** Schematic for wireless ECG measurement (A: ECG collection electrodes, B: Reference electrode, C: Bias electrode). **f** Long-term ECG signals recorded using commercial gel (top), PGEH (middle), and PGEH after 24 h (bottom). **g** Comparison of the signal-to-noise ratios of the three electrodes during long-term ECG monitoring. **h** Schematic of EMG measurement. **i** Long-term EMG signals recorded with commercial gel (top), PGEH (middle), and PGEH after 24 h (bottom). **j** Comparison of the signal-to-noise ratios of the three electrodes during long-term EMG monitoring

PGEH sensors have the ability to monitor electrophysiological signals—such as ECG and EMG signals—thereby assisting in the diagnosis of potential cardiovascular and muscle-related diseases. A low interface impedance is crucial for obtaining high-quality bioelectrical signals [60]. To this end, we developed an electrode for the acquisition of physiological electrical signals, comprising a silver ink-printed circuit and PGEH electrode (Fig. 5a). As shown in the Nyquist plot in Fig. 5b, the PGEH resistance and reactance are lower than those of the commercial Ag/AgCl electrode (310 ohms). This superior performance could be attributed to the excellent conductivity of the incorporated liquid metal nanoparticles [61]. The conductivity of the PGEH increased gradually, reaching its optimum at 0.6 wt%. However, an excess of liquid metal particles could exacerbate agglomeration, leading to a decrease in conductivity. Similarly, the Bode plot demonstrated a comparable trend (Figs. 5c and S20), whereas the impedance value of the commercial Ag/AgCl electrode at the skin interface was an order of magnitude higher. At 100 Hz, the impedance of the PGEH was 71.2 ohms, which is significantly lower than commercial electrodes, owing to poor contact between the gel and the skin.

Additionally, electrochemical impedance spectroscopy testing of the PGEH sensor on the temperature-sensing hydrogel coating was conducted at varying temperatures (Fig. 5d). Within the 0.1–100 Hz frequency range, an increase in ambient temperature from 25 to 40 °C corresponded to a rise in the impedance value from 1.96 to 4.52 S m^−1^. As epidermal physiological electrical sensors, enhanced carrier mobility and reduced interfacial resistance at elevated temperatures are crucial for improving the efficiency and accuracy of electrophysiological signal recording [62]. However, pure gelatin hydrogels can be susceptible to liquefaction near the body temperature of 37 °C, which can result in leakage at the electrode site (Fig. S21).

The unparalleled advantages of the PGEH in electrochemical performance position it as an effective epidermal sensor for monitoring electrophysiological signals—such as ECG and EMG signals—which are crucial for diagnosing potential cardiovascular and muscle-related diseases [63, 64]. Figure 5e shows the PGEH sensor affixed to the left chest of a subject to record the ECG signals, yielding a high-quality ECG signal with distinctly identifiable PQRST waveforms (Fig. S22) [49]. Remarkably, as shown in Fig. 5f, even after 24 h of continuous wear, the performance of the PGEH sensor remained comparable to that of new commercial Ag/AgCl electrodes.

Moreover, Fig. 5g shows that after 14 days of repeated testing, the PGEH achieved an average SNR of 25.2 dB, producing a high-quality ECG signal that surpassed commercial electrodes. By contrast, the SNR of the latter declined considerably after just 12 h of use, underscoring the relevance of PGEH sensors for monitoring postoperative rehabilitation exercises and treating affected muscles. Consequently, PGEH epidermal sensors exhibit enormous potential in human health monitoring and disease prediction applications. As shown in Fig. 5h, the PGEH electrode was attached to the right arm of a volunteer to detect the EMG signals. Figure 5i shows that the EMG signal was distinctly captured during the fist-clenching movement with an SNR of 17.80 dB, which was considerably higher than the SNR of 10.17 dB achieved by the commercial electrodes. Notably, the proposed system maintained high signal accuracy even under minor vibrational interference conditions (Fig. S23). Furthermore, the PGEH electrodes demonstrated long-term stability in recording high-quality EMG signals over a 24-h period. As depicted in Fig. 5j, after 14 d of continuous use, the SNR was documented as 13.1 dB, exceeding the capabilities of the commercial Ag/AgCl electrode. Additionally, biocompatibility properties are also of paramount importance. As shown in Fig. S24, compared to the control group, the number of cells cultured with the PGEH did not exhibit major changes, with over 98% of the cells in the PGEH group remaining viable, indicating the absence of cytotoxicity. Notably, rigorous testing under continuous rinsing conditions for 30 min (Fig. S25) demonstrated that neither the PGEH matrix nor the integrated sensor exhibited detectable leakage phenomena. These collective findings substantiate the structural integrity of PGEH and its environmental safety profile, suggesting promising potential for practical applications. Compared with previously reported conductive hydrogels (Table S4), the PGEH simultaneously exhibited high signal quality, low impedance, and long-term stability, making it capable of maintaining high-quality electrical signal transmissions. Consequently, epidermal sensors based on the PGEH demonstrate considerable promise in the realms of human health monitoring and disease prognosis applications.

### PGEH Sensors in EEG Collection and Attention Assessment Based on Machine Learning

**Fig. 6:**
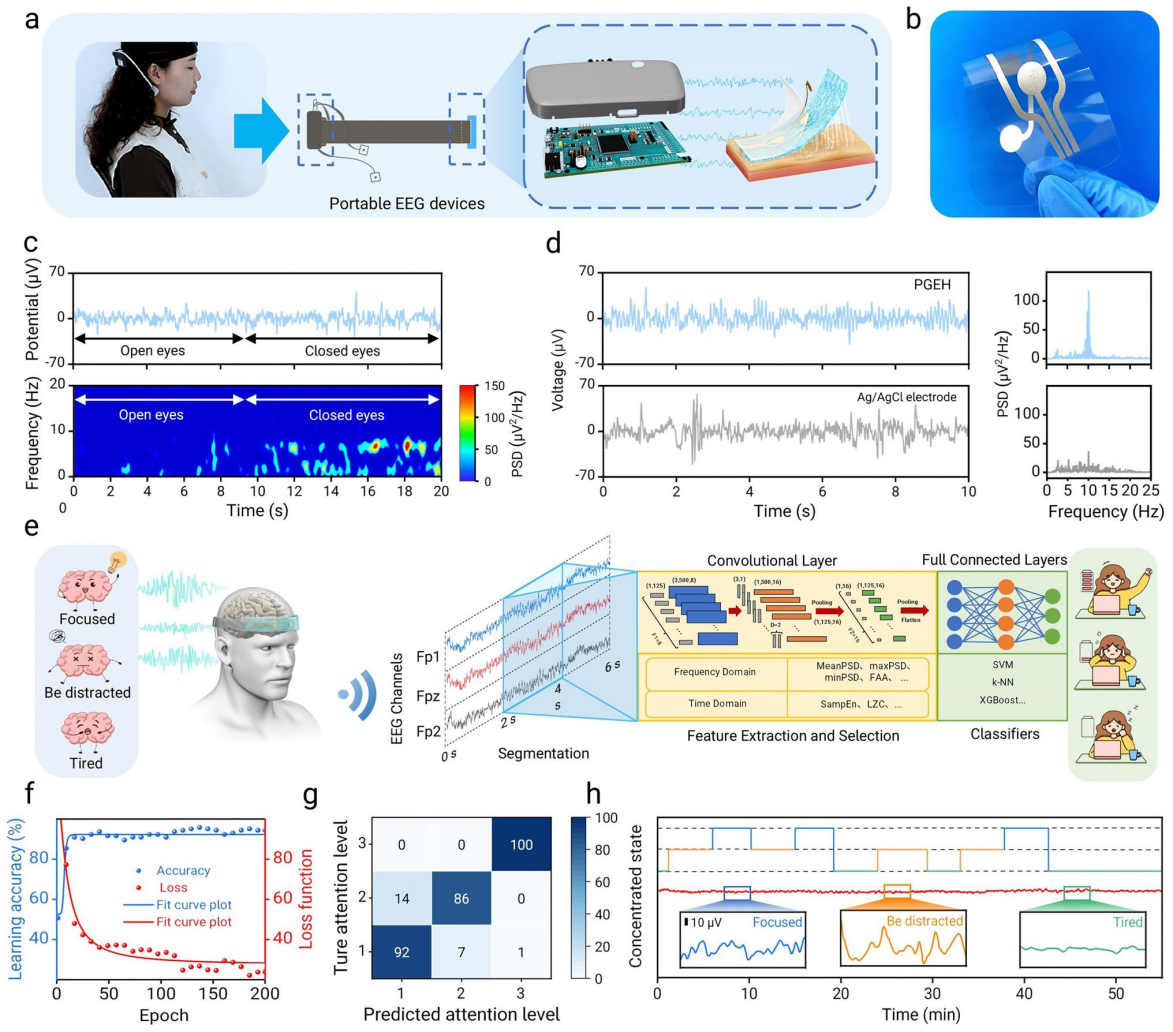
The PGEH sensors in EEG collection and attention assessment based on machine learning. **a** Schematic of an EEG collection headband for EEG monitoring, designed as a headband incorporating PGEH electrodes and Ag ink-printed circuit. **b** Image of Ag printed circuit on a polyurethane substrate. **c** EEG signals recorded using PGEH electrodes under eyes-open/eyes-closed conditions; the corresponding spectrogram shows a clear alpha rhythm in the eyes-closed state (bottom). **d** Comparison of EEG recordings from the PGEH and commercial electrodes after 6 h with the corresponding spectrum. **e** Schematic of the attention evaluation using EEG signals collected based on the PGEH through the EEGNet machine learning framework. **f** Learning accuracy and loss function over 200 iterations and corresponding **g** confusion matrix. Labels 1, 2, and 3 correspond to the focused attention state, distracted state, and rest state, respectively. **h** Long-term EEG signal acquisition for attention evaluation (inset shows the EEG signal collected in each state)

In comparison with the ECG and EMG signals, accurately recording EEG signals has consistently posed a challenge [15]. This difficulty arises because EEG signals are extremely weak—typically at the microvolt level—and can be easily affected by external factors. Additionally, complex multipoint data collection often increases the intricacy of the monitoring process, while limited collection sites make capturing high-fidelity EEG signals more difficult. To overcome these problems, we developed a flexible, portable EEG collection headband with three channels (Figs. 6a and S26). By affixing the PGEH electrode patch to the subject’s forehead, the collected EEG signals could be wirelessly transmitted to a graphical user interface, facilitating real-time monitoring and analysis (Figs. S27 and S28). Moreover, using soft polyurethane materials and an adjustable structure ensured that prolonged wear did not result in discomfort.

As shown in Figs. 6b and S29, multiple PGEH sensors (234 μm thickness) were positioned on a polyurethane (PU) flexible substrate printed with low-resistance silver paste, which offered high sensitivity and excellent skin adaptability, effectively minimizing motion interference and enhancing signal collection accuracy, with noise power as low as 7.3 μV^2^. Figure 6c shows the EEG signals recorded under eyes-open and eyes-closed conditions, with the corresponding time–frequency spectrograms indicating the absence of an alpha rhythm (8–13 Hz) in the eyes-open condition, whereas a distinct alpha rhythm is evident in the eyes-closed condition. Compared to the commercial electrodes (Fig. 6d), the PGEH sensors could record long-term, high-fidelity alpha rhythms (10 Hz) during the signal collection process and provide continuous monitoring for up to 48 h (Figs. S30 and S31), whereas the signal quality of commercial electrodes declines after six h of use. In addition, PGEH can also be applied to the EEG signal acquisition in various movements, such as eyes blinking (Fig. S32), head shaking, and the analysis of visual evoked potential (Fig. S33). The advantages of the PGEH electrodes in EEG signal collection are self-evident. Their high-fidelity, long-term stability, and comfort suggest enormous potential for development in the fields of clinical brain medicine and scientific research.

Attention assessment and feedback technology based on EEGs is increasingly gaining attention in both research and practical applications [65, 66]. In this work, we combined high-fidelity EEG signals collected via a three-channel EEG collection headband with advanced machine learning algorithms to achieve precise classification and identification of attention levels. As shown in Fig. 6e, the EEG-based attention assessment experiment proceeded through several sequential steps—that is, reaction force testing, EEG recording, signal preprocessing, feature extraction, model training, and validation [67]. During the experiment, participants wore headphones to eliminate auditory interference and were instructed to maintain a relaxed sitting posture (Fig. S34). As shown in Fig. S35, we used three indicators to assess participants’ concentration—namely, color, shape, and pattern orientation. Each experiment commenced with participants focusing on the monitor and was conducted under three conditions—that is, focused state, distracted state, and exhaustion state conditions.

An EEG headband device recorded signals from three channels. As shown in Fig. 6f, we collected data from 20 healthy subjects, including 10 males and 10 females, with an average age of 25 ± 3 years. All participants confirmed that they had not taken any psychotropic drugs within 24 h prior to the experiment. Tenfold cross-validation was performed using all the data. We selected multiple machine learning models for attention classification [68–70], where the input to EEGNet was the preprocessed EEG signal, and the inputs to other traditional machine learning models, for example, support vector machines (SVM), k-nearest neighbors (KNN), extreme gradient lifting (XGBoost), were EEG feature data, including PSD (power spectral density), FAA (frontal alpha asymmetry), etc. [71]. We set the network parameters for EEGNet based on the task (SI. Model Structures of EEGNet). By comparing the performance of different classifiers, we found that the training loss of EEGNet gradually decreased to 0.021 after 200 epochs (Fig. 6g), with a tenfold average accuracy reaching 91.38%. In contrast, the performance of other classifiers was significantly affected by feature engineering and achieved notably lower accuracy—approximately 20% lower than EEGNet (Fig. S36). The high-performance EEGNet can be lightweight and has the potential for onboard deployment, which facilitates the ubiquity of EEG-based devices. Additionally, as shown in Fig. 6h, we evaluated subjects’ performance across varying attention levels over an extended period. The results indicated that during the focused state, the EEG exhibited higher activity, followed by the attention-disturbed state, whereas the fatigued state demonstrated the lowest activity levels. This variation in activity was evident not only in the amplitude of the waveform but also in its frequency distribution. In summary, through the integration of an EEG collection headband and machine learning algorithms, long-term monitoring of users’ brain waves could be achieved. This allowed for a systematic analysis of attention patterns and fatigue cycles, thereby assisting users in developing more effective work and rest plans (Fig. S37).

## Conclusions

In this work, we developed hydrogel-based sensors suitable for wearable high-performance epidermal sensing, encrypted messaging, and attention assessment applications. Using the molecular interactions between gelatin and acrylic polymers, combined with the superior conductivity of liquid metal, we endowed the PGEH with intelligent adhesion properties, allowing for painless removal and minimizing secondary damage to delicate tissues. Additionally, the PGEH could be assembled into a multifunctional epidermal sensor capable of sensitively monitoring both large human movement signals and small electrophysiological signals, such as ECG, EMG, and EEG signals. This capability provides valuable information for limb rehabilitation training and cardiovascular disease diagnosis applications. Moreover, machine learning-assisted human attention assessment based on the collected EEG signals was implemented, featuring a high-precision classification function for attention states. With its outstanding performance and versatility, the PGEH sensor could play a major role in various fields and lead a paradigm shift in the next generation of wearable electronic skin, rehabilitation medicine, and machine learning-assisted human–computer interaction. This research could broaden the development trajectory of epidermal electronics.

## Supplementary Information

Below is the link to the electronic supplementary material.

Supplementary file1 (MP4 2651 KB)

Supplementary file2 (MP4 1492 KB)

Supplementary file3 (MP4 1405 KB)

Supplementary file4 (MP4 2491 KB)

Supplementary file5 (DOC 30497 KB)
