# Supplementary material for: Machine Learning Enabled Reusable Adhesion, Entangled Network-Based Hydrogel for Long-Term, High-Fidelity EEG Recording and Attention Assessment
Source: Nanomicro Lett. 2025 May 29;17:281. doi: 10.1007/s40820-025-01780-7 (PMC12122957; doi:10.1007/s40820-025-01780-7)
Supplement: Supplementary file 5 — Supplementary file5 (DOC 30497 KB) [file 40820_2025_1780_MOESM5_ESM.doc]

Supporting Information for

**Machine Learning Enabled Reusable Adhesion, Entangled Network Based Hydrogel for Long-Term, High-Fidelity EEG Recording and Attention Assessment**

Kai Zheng^1^, Chengcheng Zheng^1^, Lixian Zhu^1^, Bihai Yang^1^, Xiaokun Jin^1^, Su Wang^1^, Zikai Song^1^, Jingyu Liu^1^, Yan Xiong^2^, Fuze Tian^3^*, Ran Cai^1^*, Bin Hu^1^*

^1^ Key Lab of Brain Health Intelligent Evaluation and Intervention, Beijing Institute of Technology, Beijing 100081, P. R. China

^2^ Analysis & Testing Center of Fangshan District, Beijing Institute of Technology, Beijing 100081, P. R. China

^3^ School of Information Science and Engineering, Lanzhou University, Lanzhou 730000, P. R. China

* Corresponding author. E-mail: [**bh@bit.edu.cn**](mailto:bh@bit.edu.cn) (Bin Hu); [**cairan@bit.edu.cn**](mailto:cairan@bit.edu.cn) (Ran Cai); [**tianfz17@lzu.edu.cn**](mailto:tianfz17@lzu.edu.cn) (Fuze Tian)

S1 Characterization of the PGEH

S1.1 Experimental Equipment

Fourier transform infrared spectroscopy (FTIR) was tested using a Fourier transform infrared spectrometer (FTIR, Nicolet iS10, Thermo Fisher Scientific Co. Ltd.) with a scanning range of 400-4000 cm^−1^. X-ray photoelectron spectroscopy (XPS) measurements were measured on a K-Alpha X-ray Photoelectron Spectrometer (Thermo Scientific, USA). The surface morphology was observed by an optical microscope (Olympus, Japan) and scanning electron microscopy (SEM, JSM-7500F, Joel Co. Ltd, Japan) at an accelerating voltage of 5 kV. The SEM specimen was sputter-coated with gold.

**S1.2 Mechanical Properties Test of the PGEH**

All mechanical tests were carried out atroom temperature (20 °C)on an electronic tensile machine (Model ES30, Mark-10 Instrument Co. Ltd.). PGEH of dimensions 25 mm×10 mm×1 mm was used to assess the successive stress–strain curves using tensile machine at a stretching speed of 100 mm·min^-1^. For compression measurements, cylindrical hydrogels of diameter 10 mm and height 12 mm were subjected to continuous loading–unloading cycles at a constant rate of 10 mm·min^-1^. For the lap-shear testing, samples were prepared by adhering two substrate pieces with thin square hydrogels of dimensions 10 mm long, 15 mm wide, and 0.5 mm thick, resulting in an overlapping area of 15 cm². Prior to testing, a 10 s pressing at 100 g was applied, and all tests were conducted at a constant tensile speed of 10 mm·min^-1^. and rheological characterization of the PGEHs was conducted using a rheometer (MCR 72, Anton Paar). For amplitude sweeps, a 12 mm parallel plate geometry was employed with a 1 mm gap size, at a temperature range of 25–50 °C and a strain of 10%.The adhesive properties of the hydrogels were evaluated using the standard lap-shear test and conducted on Mark-10 equipped with a 2 kN load cell.

**S1.3 Electrical Properties Test of the PGEH**

Electrochemical impedance spectroscopy measurements were conducted using an electrochemical workstation (760E, CHI) with a sweep range from 0.1 Hz to 100 kHz at an open-circuit potential of 0.1 V. A hydrogel of 8 mm length, 8 mm width, and 2 mm thickness was placed between two zinc sheets (10 × 20 mm). The interfacial contact impedance was measured using a distance of 13 mm between each electrode pair. All impedance measurements were conducted at an amplitude of 100 mV.

**S1.4 Physiological electrical signal acquisition for the PGEH**

The EMG signals were obtained byadhesive PGEHs electrodes and commercial electrodes (Ag/AgCl), incombination with the biosignal acquisition system (OpenBCI, USA).

The monitoring of EEG signals was realized primarily by a device consisting of electrodes, sensors, an acquisition card, and software (Detailed information is mentioned in S26-28). The signal quality was measured using the SNR, a high SNR indicating that the signal power was greater than the noise power, resulting in higher fidelity. It could be calculated as follows:


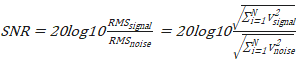
 (S1)

where *Vsignal* and *Vnoise* denote the voltage values of the signal and noise, respectively, and *N* denotes the total number of samples.

**S1.5 Cytocompatibility investigation**

The cytotoxicity of PGEHs was measured using a leaching pattern test on L929 cells. Initially, the PGEHs were sterilized via UV irradiation, soaked in DMEM for 24 hours at 37 °C, and then filtered to obtain the extract solutions. Subsequently, L929 cells were seeded in a 96-well plate at a density of 7.0 × 10^3^ cells/well and cultured in an incubator at 37 °C with 5% CO2, using 100 μL of hydrogel extract and growth media, respectively. At intervals of 24, 48, and 72 hours post-culturing, CCK-8 solution (100 μL) was introduced into the medium of each well and co-cultured for 2 hours. Following this, the OD450 values of the medium were measured using a microplate reader (SPARK 10M, TECAN). Lastly, the cells were stained with Calcein-AM and observed under a laser confocal microscope (Olympus, FV1200).

**S1.6 Electroencephalogram Data Processing and Feature Extraction**

EEG signals were recorded by the EEG sensor at a sampling frequency of 250 Hz. A bandpass finite impulse response (FIR) filter with a passband of 0.5-45 Hz was applied to filter the raw EEG signals, removing baseline drift and high-frequency interference (such as power line interference and radio-frequency interference). Since the electrodes were placed near the eyes, ocular artifacts (OAs) were inevitably collected. Therefore, we used a model combining discrete wavelet transformation (DWT) and Kalman filtering to remove OAs. This model consists of four steps: (i) detecting the OA regions using a threshold determined by the global mean and variance of the EEG data; (ii) performing DWT on the marked OA regions from step one to extract approximate OAs; (iii) applying a Kalman filter to the extracted approximate OAs to further remove EEG information contained in the extracted signals and obtain pure OAs; (iv) subtracting the optimized OAs from the original EEG. Although no additional feature extraction was conducted prior to inputting the data into the model, analyzing the EEG data required calculating the power spectral density (PSD) across the full frequency band to evaluate the signal characteristics. The PSD was computed using the Welch method [S1], which involves dividing the signal into several overlapping windows. For each window, the fast Fourier transform (FFT) of the signal segment is calculated, followed by averaging the PSD of all windows. Specifically, the frequency domain representation of each signal segment is expressed as follows:


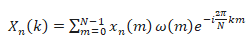
 (S2)

where *N* = 100 represents the number of data points in each window, and
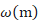
 is the window function. The resulting PSD for each segment is as follows:


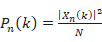
 (S3)

The power spectral densities of all windows are averaged to obtain a complete *PSD* estimate:


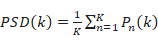
 (S4)

where *K* denotes the total number of overlapping windows used across the signal.

**S1.7 Machine learning for EEG Sustained Attention recognition**

In this test, the informed written consent was signed by all participants prior to the research. The participants are required to identify pattern features such as color, shape, and direction. Various patterns are presented around a fixation point, and subjects must quickly locate the correct pattern. Upon identifying the correct pattern, participants should press the 'Z' key. If no correct pattern is visible, they should press the '/' key on the keyboard. Each group of patterns appears at 500 ms intervals, consisting of 10 trials per group. Subjects repeat the test over 8 groups, following a practice session conducted beforehand. At the end of each set of ten trials, the screen displayed the accuracy for that group. This method reduces the reliance on extended response times and high refresh intervals, which can cause fatigue and reduce measurement precision. By incorporating a shorter interval and a binary decision-making task, this approach provides a more dynamic assessment of sustained attention. Correct responses under normal conditions correspond to the focused attention state [S2], while incorrect responses when strong, distracting noise is played through headphones correspond to the distracted state [S3]. Staring at a blank screen with a relaxed mind corresponds to the rest state after fatigue [S4].

**S1.8 Model Structures of EEGNet**

EEGNet [S5] is selected for its ability to effectively extract spatial-temporal features from EEG data, which is critical for accurate classification in EEG-based applications. The model structure of EEGNet takes EEG data as input, shaped as (3, 500, 1), which represents data from three channels with 500 time sampling points per channel. Initially, the model applies a two-dimensional convolution layer with a kernel size of 1×125 to extract features along the time dimension, followed by standardization through a batch normalization layer. Subsequently, a depthwise convolution layer is employed, utilizing three convolution kernels (one for each channel) with a depth multiplier of 2 to extract spatial features, by processing the features of each channel independently. After this, batch normalization is applied again, and the outputs are activated using the ELU activation function. An average pooling layer is then utilized to reduce the time dimension features by a factor of four, decreasing data complexity and mitigating overfitting. Following this, a Dropout layer with a dropout rate of 0.5 is implemented to randomly deactivate some neurons. The model then employs a separable convolution layer to further extract features, with a convolution kernel size of 1×16 that outputs 16 feature maps. After this convolution, batch normalization is applied once more, followed by activation using the ELU function. The time dimension is subsequently reduced to 1/8 through another average pooling layer. Finally, the model flattens the three-dimensional feature map into a one-dimensional vector and maps these features to the output category space via a fully connected layer. The model outputs three classification results, which are normalized using the softmax activation function. These methodologies are implemented using PyTorch.

**S2 Supplementary Figures**

**Fig. S1** **SEM images of PGEH with varying EGaIn content.** **a** Representative microscopy images show that the PGEH-0LM possesses a consistent cross-linking network. **b** Conductive EGaIn particles are distributed within the pores of PGEH. **c** High-magnification SEM image of the region marked in **b**

**Fig. S2** Element mapping of the PGEH. **a** SEM image and the corresponding element mapping. **b** C. **c** O. **d** N. **e** Ga. **f** In


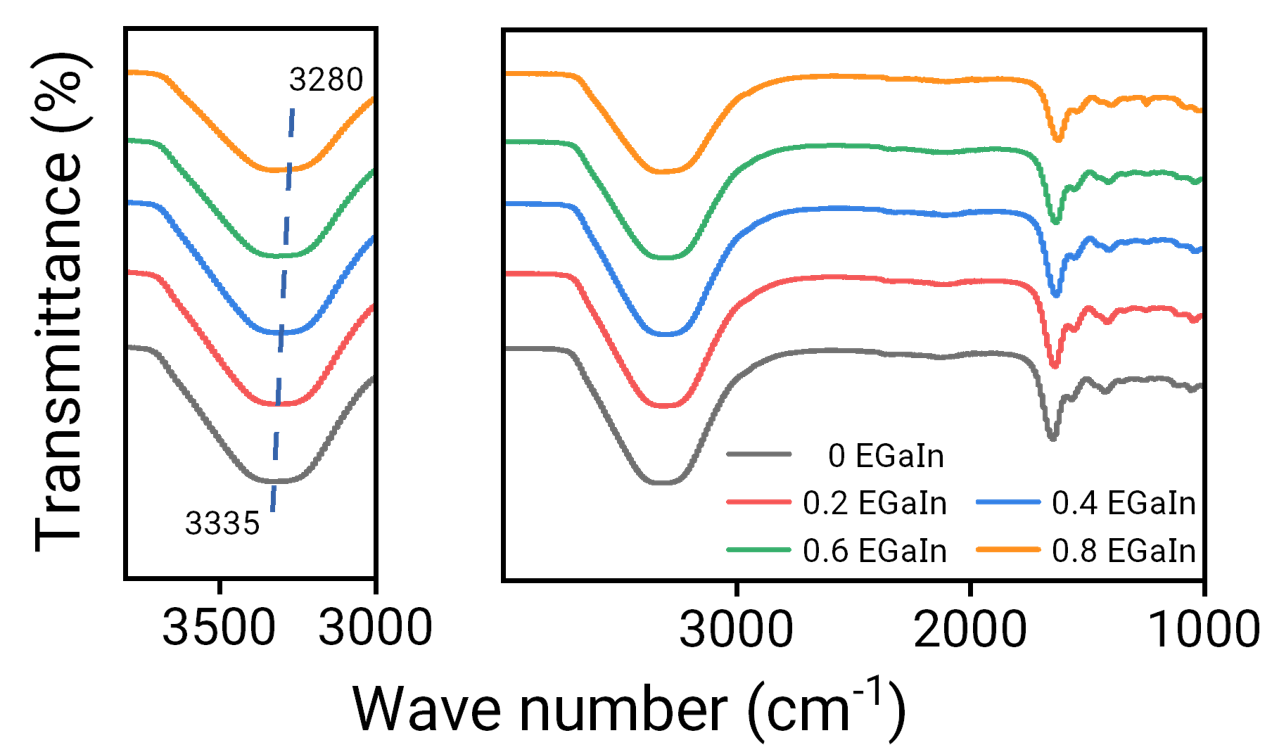


**Fig. S3** **The interaction between polymer network and EGaIn.** FTIR spectrum of the PGEH at different EGaIn contents. As the EGaIn content increases, the PGEH peak near 3300 cm^-1^ shifts leftward, moving from 3280 cm^-1^ to 3335 cm^-1^


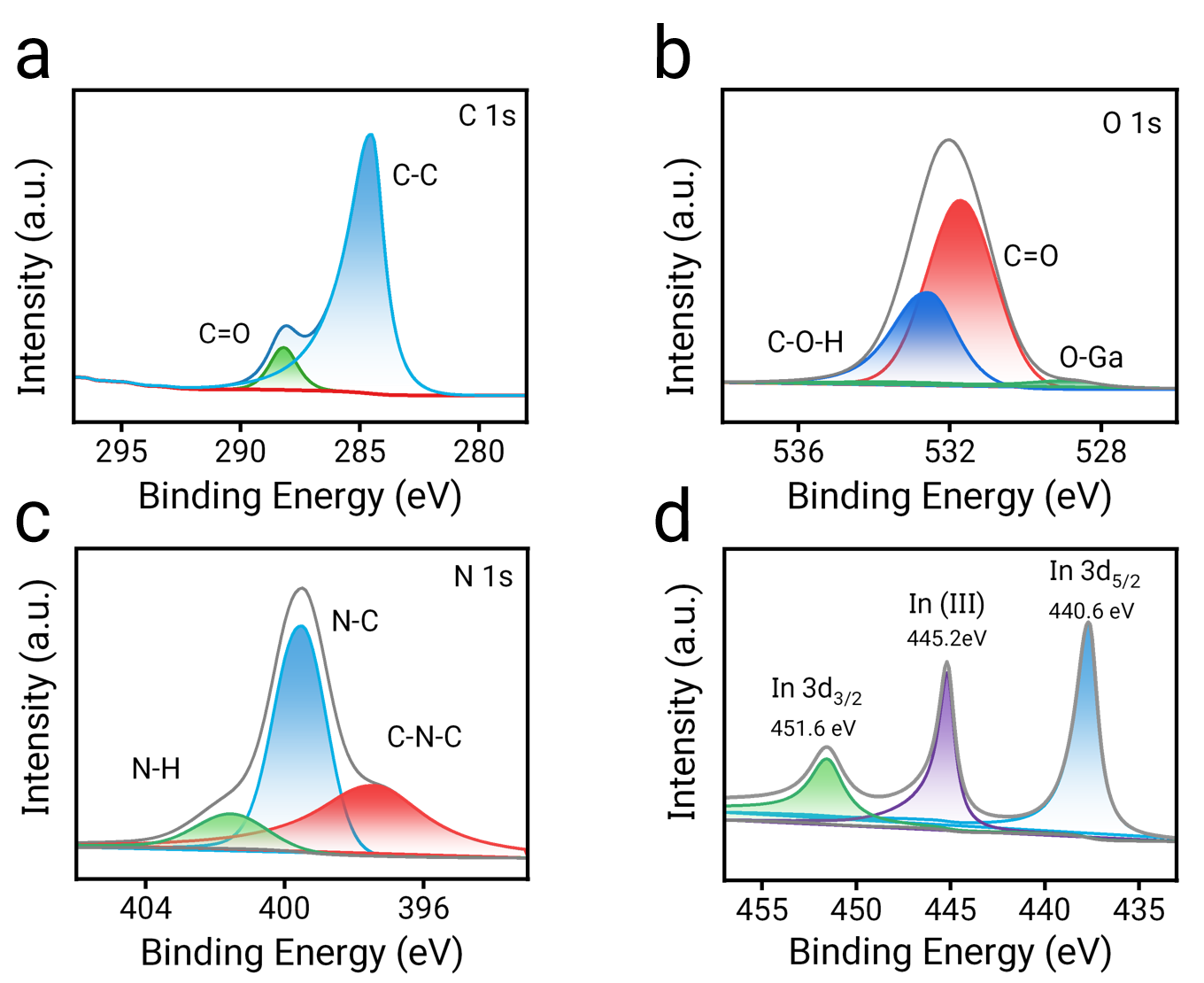


**Fig. S4 XPS spectra of the PGEH.** **a** The C 1s spectra of PGEH could be fitted by peaks at different energy levels: 284.8 eV corresponds to carbon atoms in C-C and C=O bonds, 286.3 eV to carbon atoms from C-C bonds, and 288.0 eV to carbon atoms in C=O bonds. **b** The O 1s spectra of PGEH. The main peak at 532.5 eV indicates to C=O group oxygen atoms. A satellite peak at 531.5 eV suggests oxygen in Ga-O group, and another peak at 533.5 eV suggests a C-O-H group. **c** The N 1s spectra of PGEH displays three peaks at 397.4 eV, 399.5 eV and 401.6 eV, which are derived from N–C, N–H and C–NC bonds, respectively. **d** Peaks at 443.26 eV and 450.81 eV, separated by 7.55 eV, are ascribed to the binding energies of In 3d_5/2_ and In 3d_3/2_, indicating the oxidation state of indium.


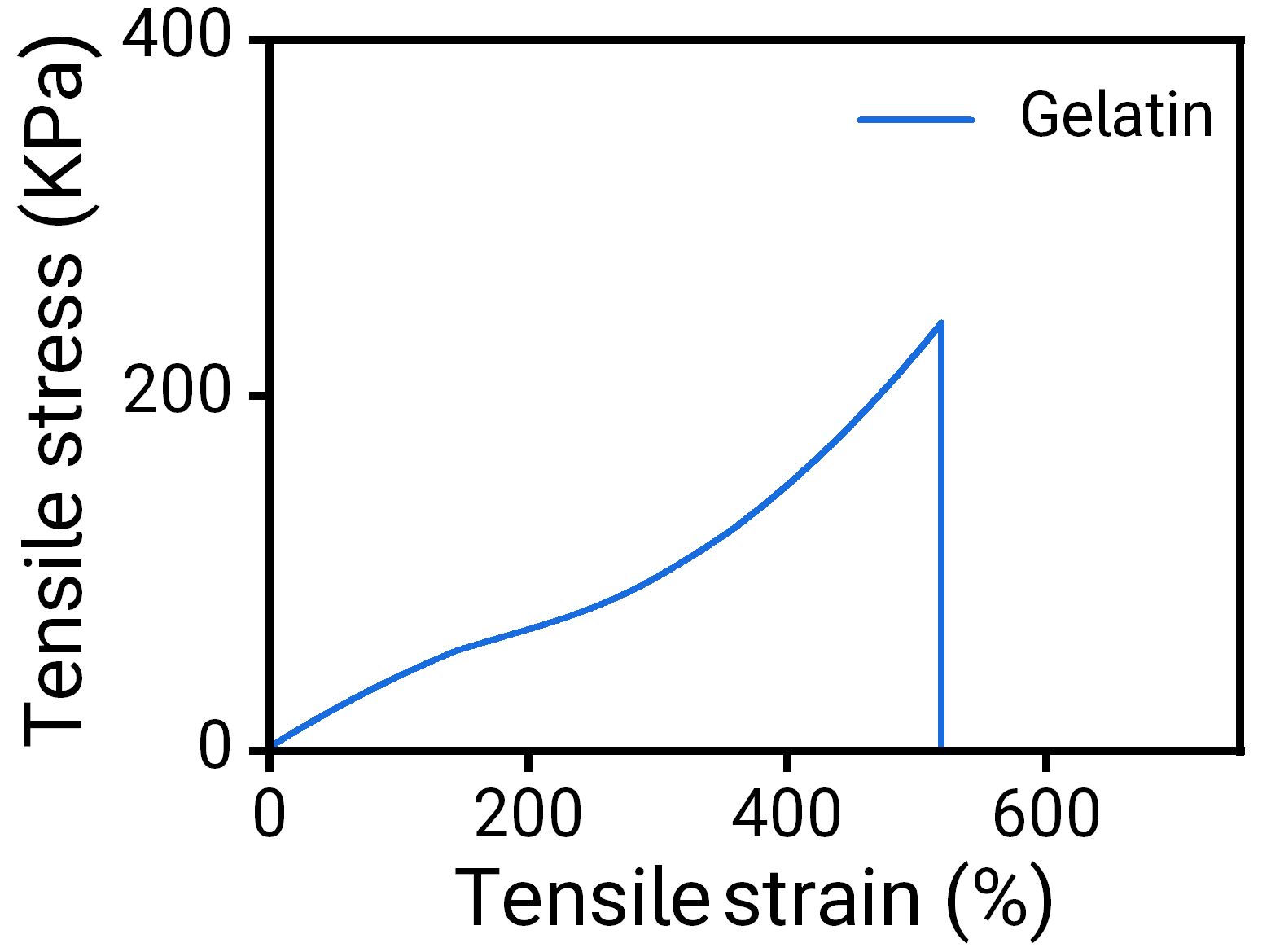


**Fig. S5 The stress-strain curves of pure gelatin hydrogel.** The pure gelatin hydrogel was tested at a tensile rate of 10 mm∙min^−1^


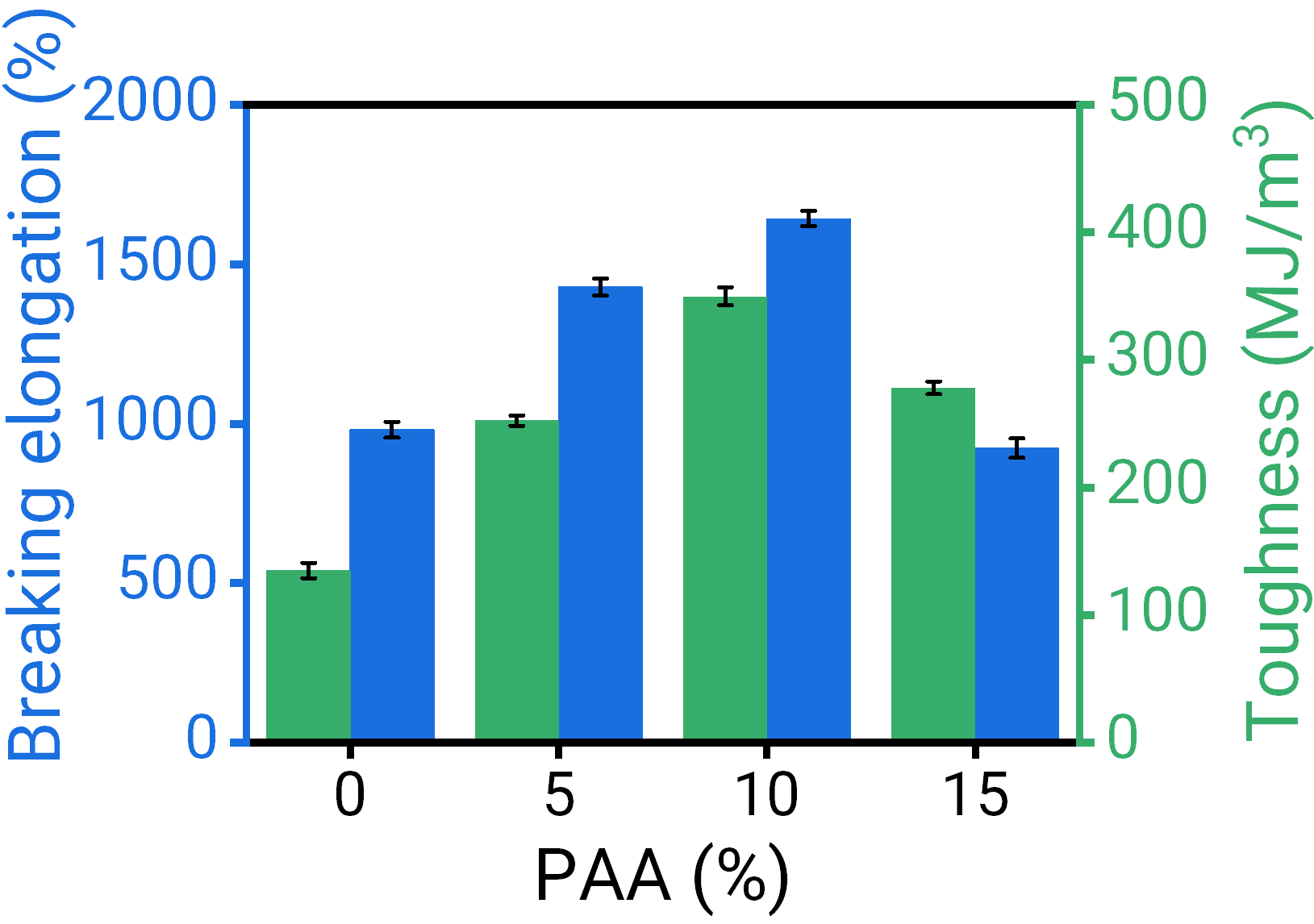


**Fig. S6 Columnar statistical diagram of the mechanical properties of PEGH hydrogel influenced by varying PAA content (Tensile experiment).** The PEGH was tested at a tensile rate of 10 mm∙min^−1^. As PAA content increased, the number of crosslinking points in PEGH also rose, enhancing toughness and elongation at break. However, excessive crosslinking can render the hydrogel hard and brittle. The optimal comprehensive performance was achieved when PAA content reached 10%. Data are reported as means ± standard deviations (SDs, N ≥ 3)


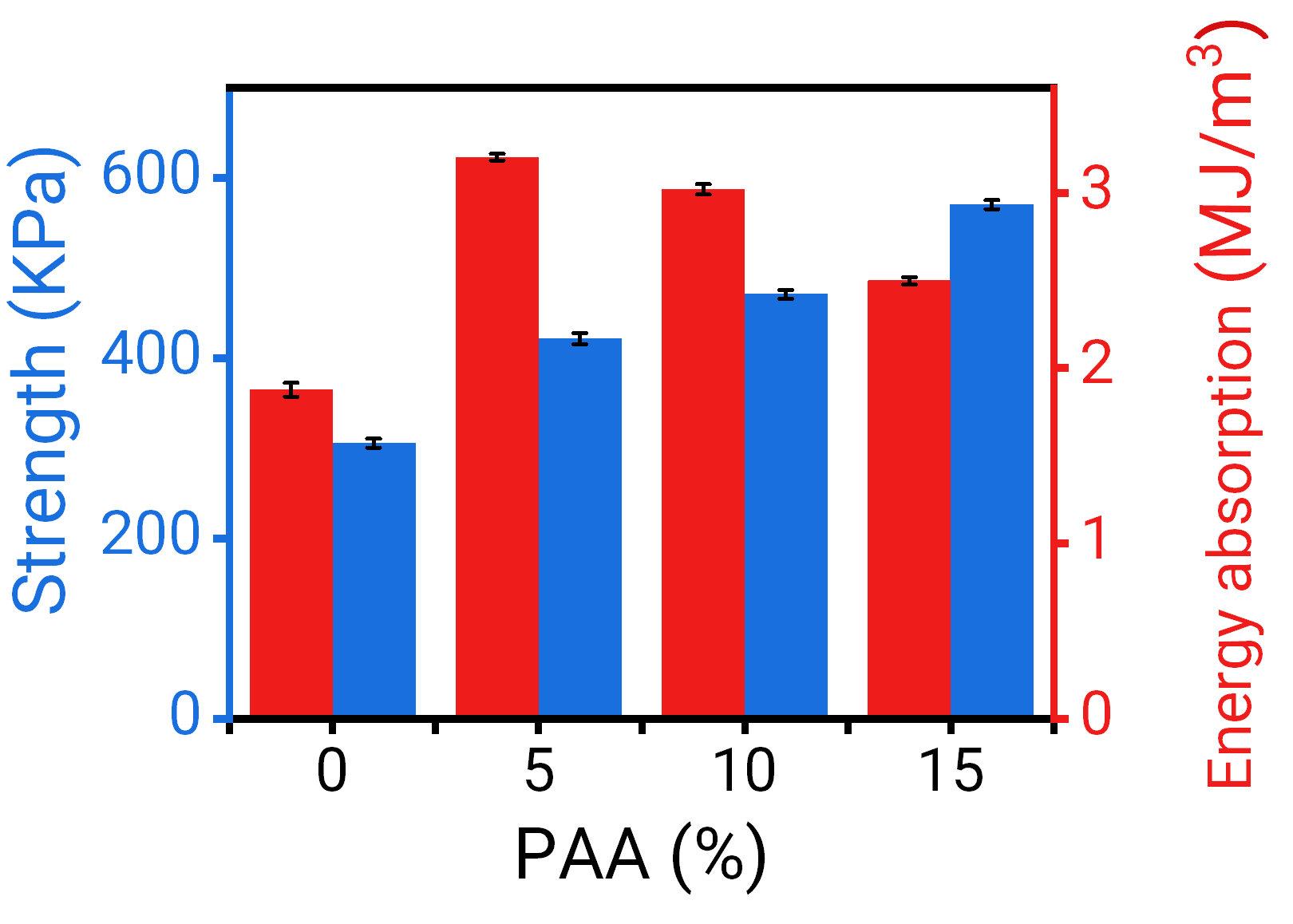


**Fig. S7 Columnar statistical diagram of the mechanical properties of PEGH hydrogel influenced by varying PAA content (Compression experiment).**The PEGH was tested at a compression rate of 10 mm∙min^−1^. For elastomers, achieving high compressive strength and low energy loss is crucial. Hence, PEGH with a 10% PAA content is the most suitable. Data are reported as their means ± SDs (N ≥ 3)


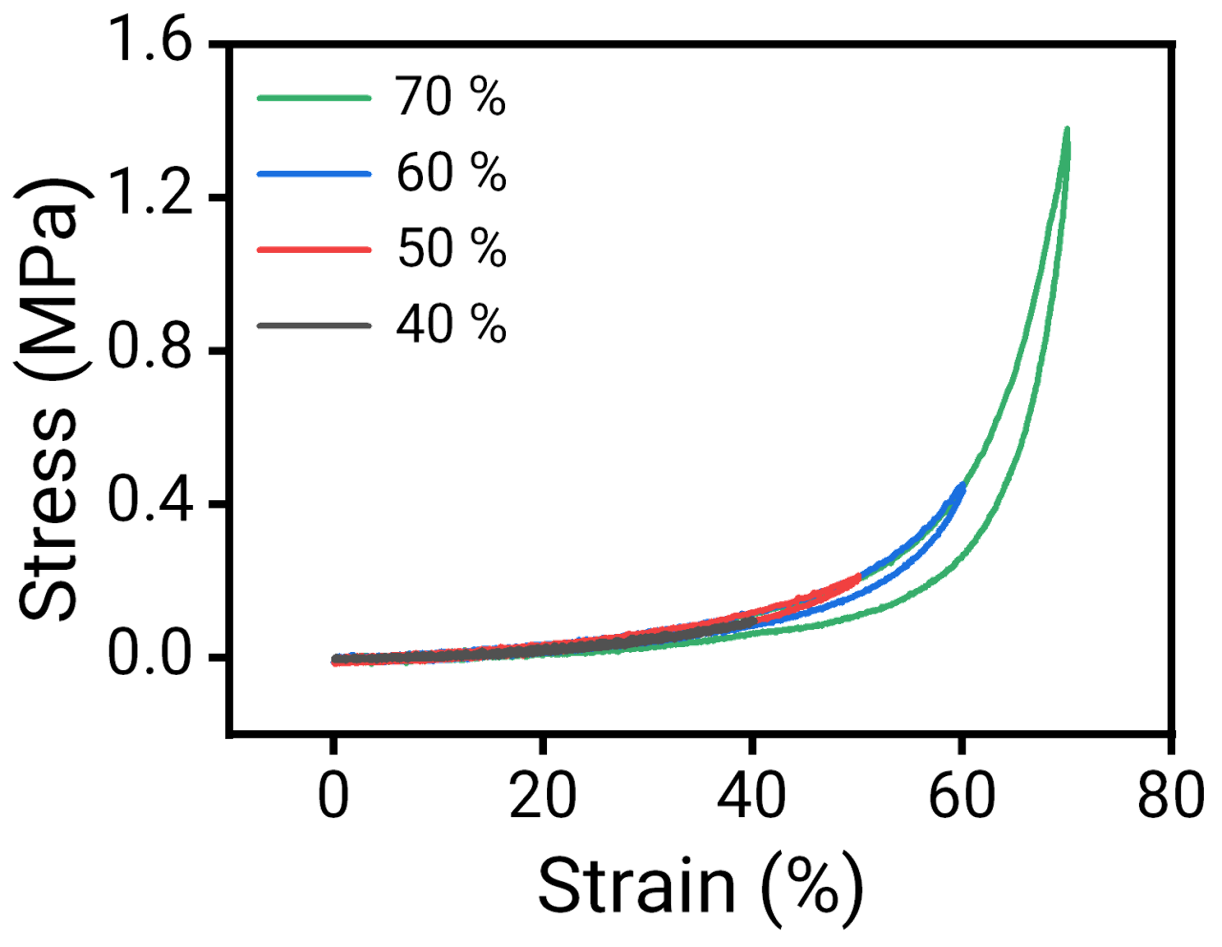


**Fig. S8 Tensile loading-unloading stress-strain curves of PGEH.** PGEH was tested under different maximum strains of 40%, 50%, 60%, and 70% (encapsulated with VHB tapes), at a tensile rate of 10 mm∙min^−1^.


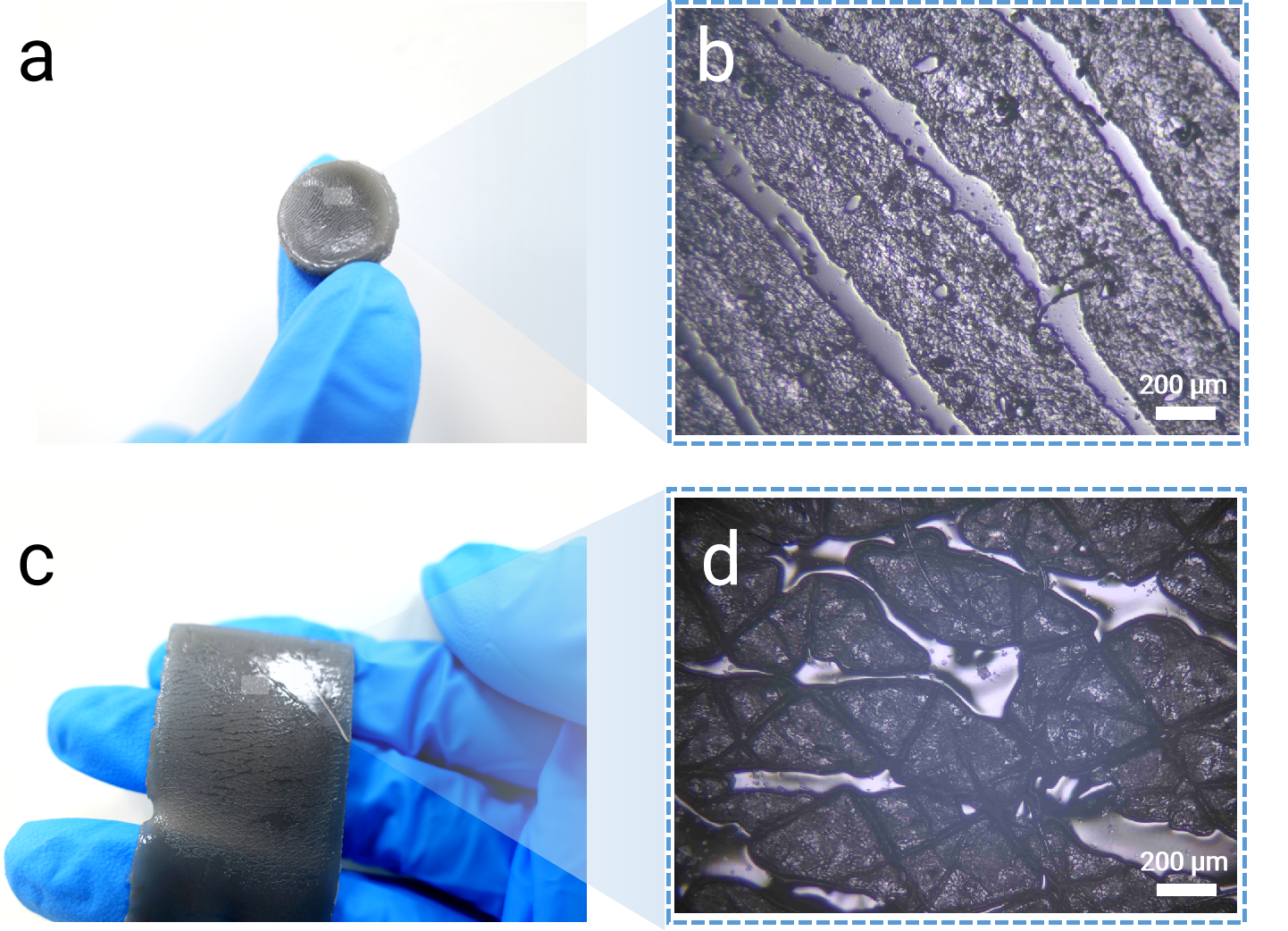


**Fig. S9 Photographs of conformal attachment between PEGH and wrinkled skin.** Upon attaching the PEGH hydrogel to the finger (a, thumb) and wrist joint (**c**), it maintained stable contact with the surface of the wrinkled tissue. This is evident from the corresponding images (**b**) and (**d**), which demonstrate that the skin's wrinkle texture is distinctly preserved under the electron microscope. This tissue-like compliance is vital for electrode, as it not only addresses the biomechanical mismatch at skin-electrode interfaces but also minimizes the effects of motion.


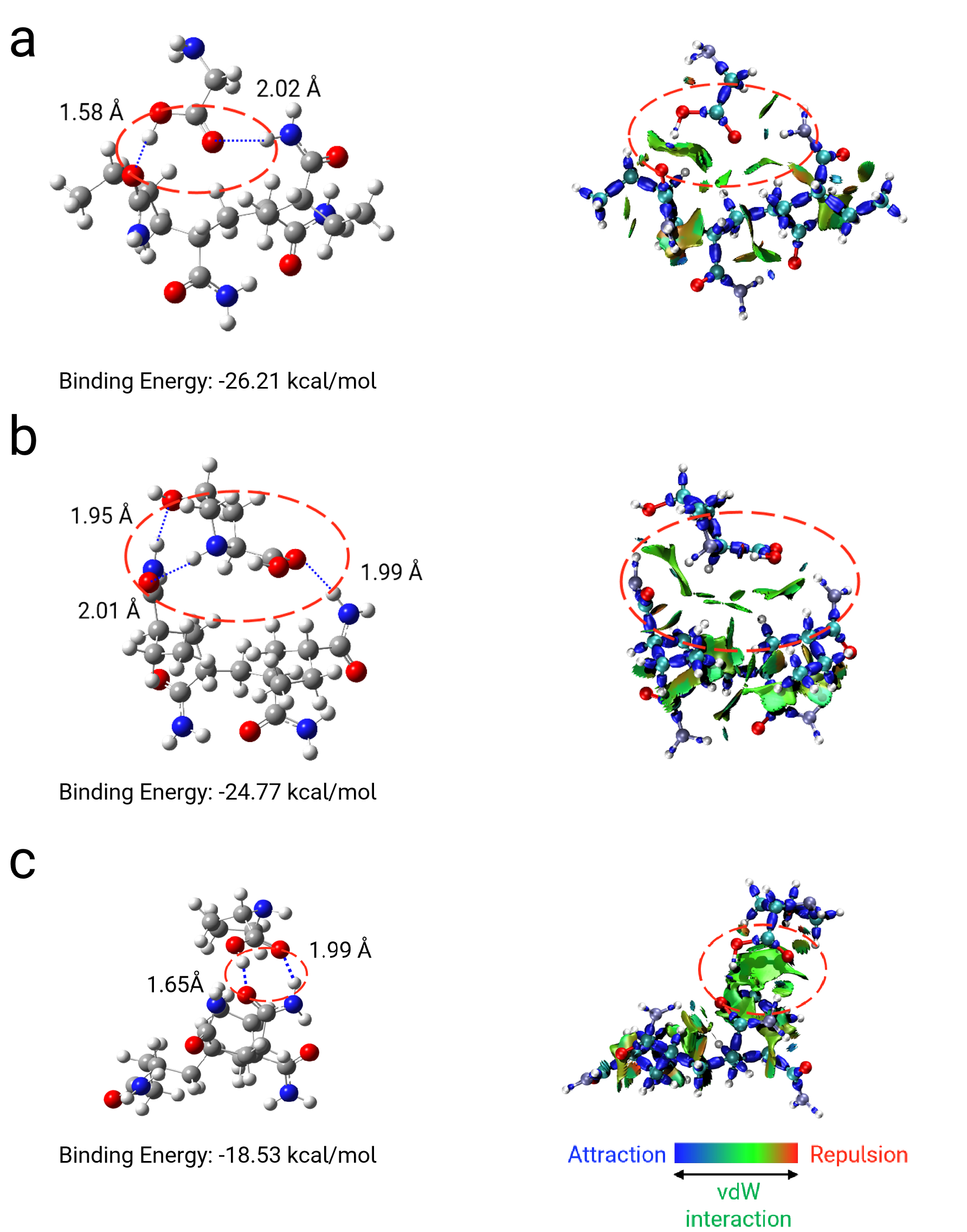


**Fig. S10 Density functional theory calculations demonstrating the interactions between PAm and glycine, hydroxyproline and proline from Gelatin chains.** Complex of (**a**) PAm-glycine, (**b**) PAm-hydroxyproline and (**c**) PAm-proline. The adsorption configuration and Interaction Range Indicators of three models. The color bar indicates the type and strength of the interaction: blue means notable adsorption, red means notable repulsion, and green indicates that Van der Waals (VDW) interactions


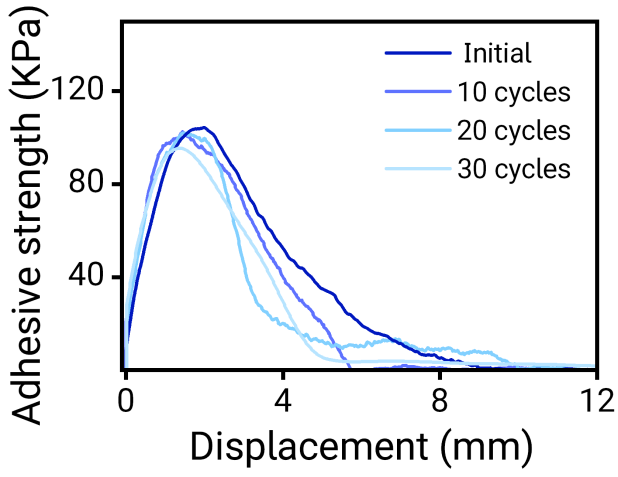


**Fig. S11 Cyclic adhesion performance testing of PGEH.** The adhesion ability of PEGH hydrogel was tested over 30 cyclic periods, and no significant decrease in adhesion force was observed


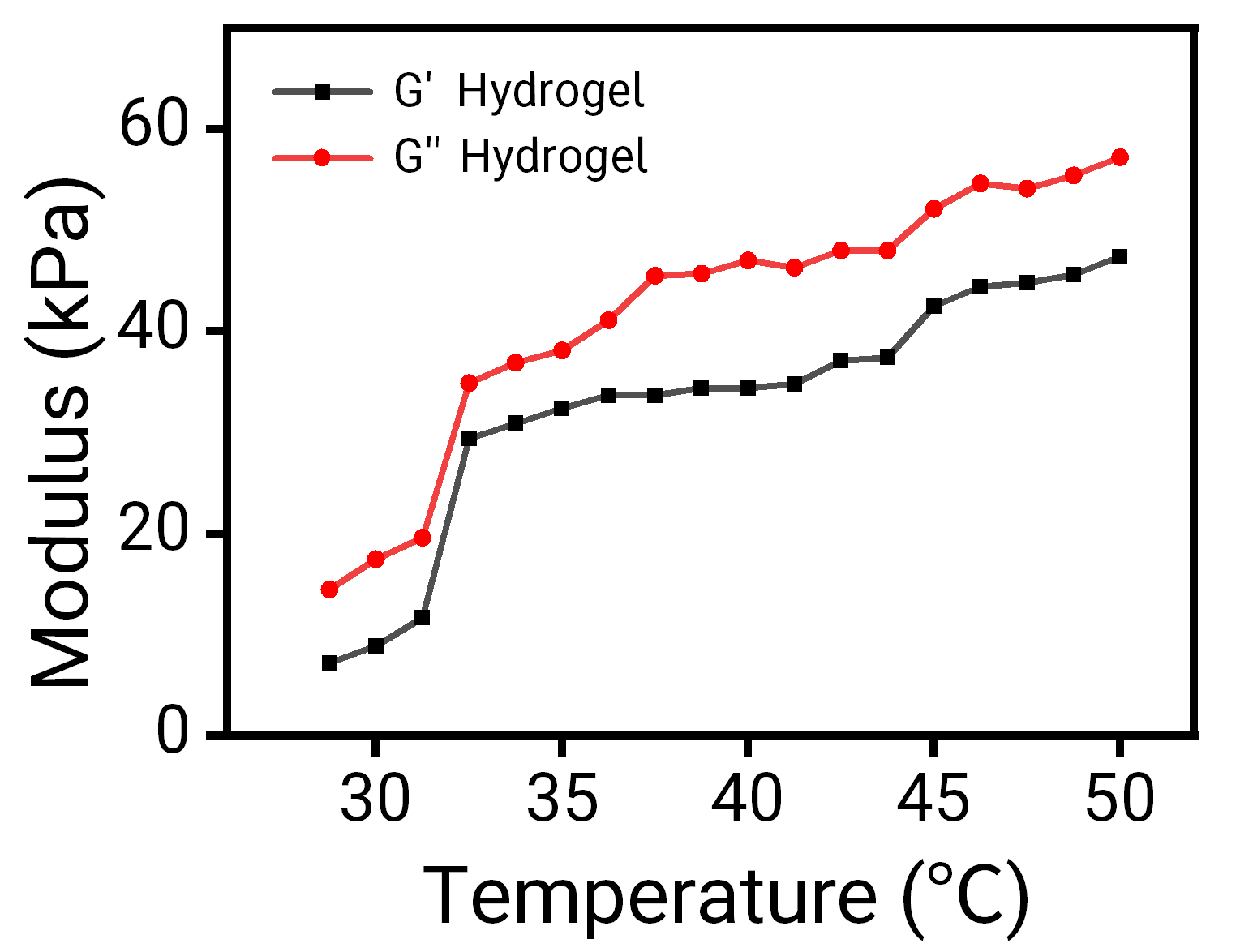


**Fig. S12 Dynamic mechanical analysis of gelatin hydrogel.** Temperature sweep showed storage modulus (G’) and loss modulus (G’’) of gelatin hydrogel between 25 ℃ and 50 ℃ under a constant strain amplitude of 1% and a frequency of 1 Hz


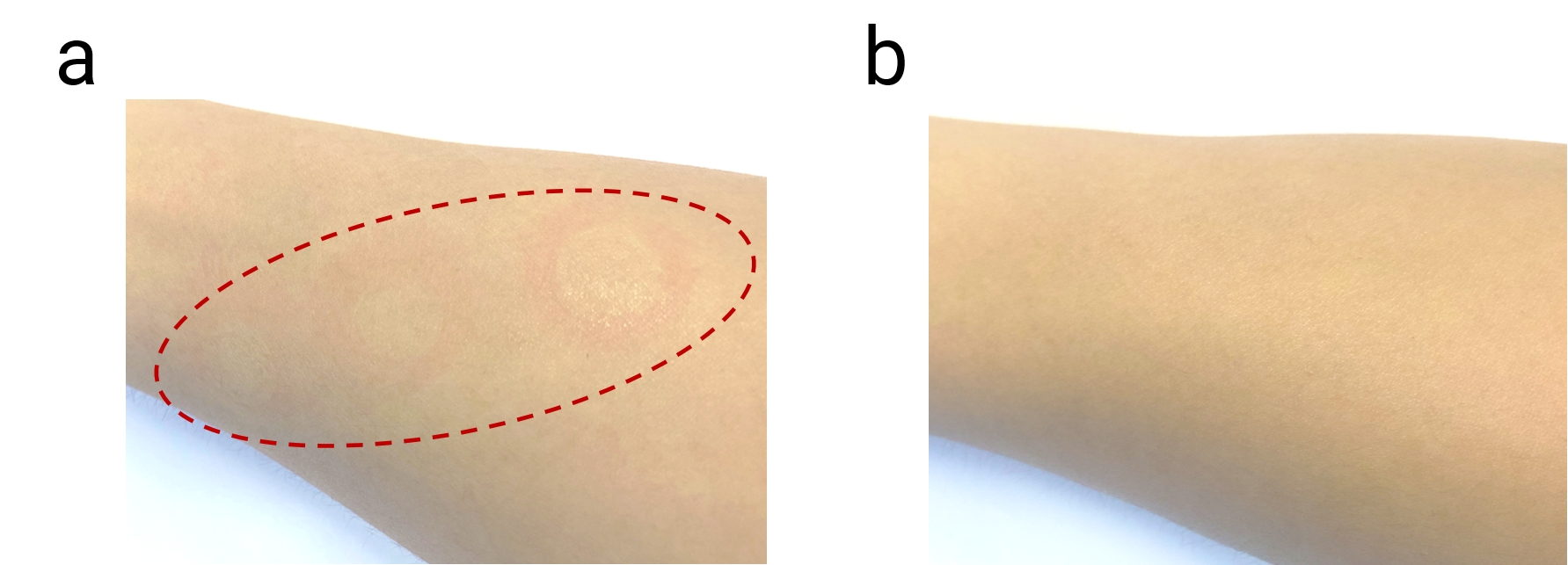


**Fig. S13 Photographs of PEGH and commercial electrodes after being peeled off from the skin.** (**a**) Commercial Ag/AgCl electrodes and (**b**) PGEH electrodes were attached to the arm and subsequently peeled off. PGEH electrodes left no trace or allergic reaction


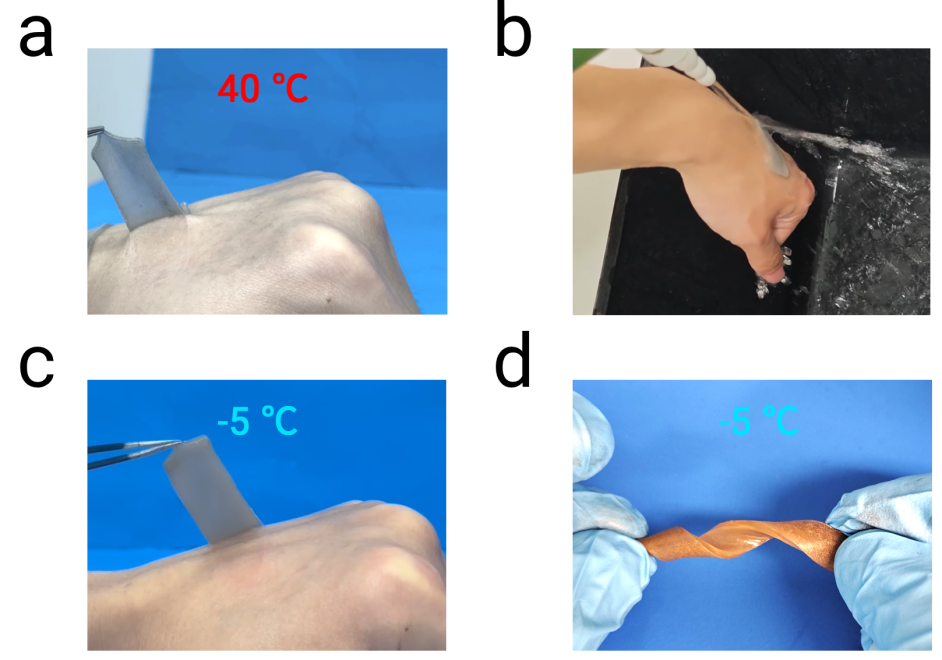


**Fig. S14.** **a** Images displaying the PGEH adhered to human skin at 40 °C. **b** Cold water rinsing tests of the PGEH. **c** Adhesive behavior of PGEH on skin at -5 °C. **d** Flexibility evaluation of the PGEH under bending deformation at -5 °C


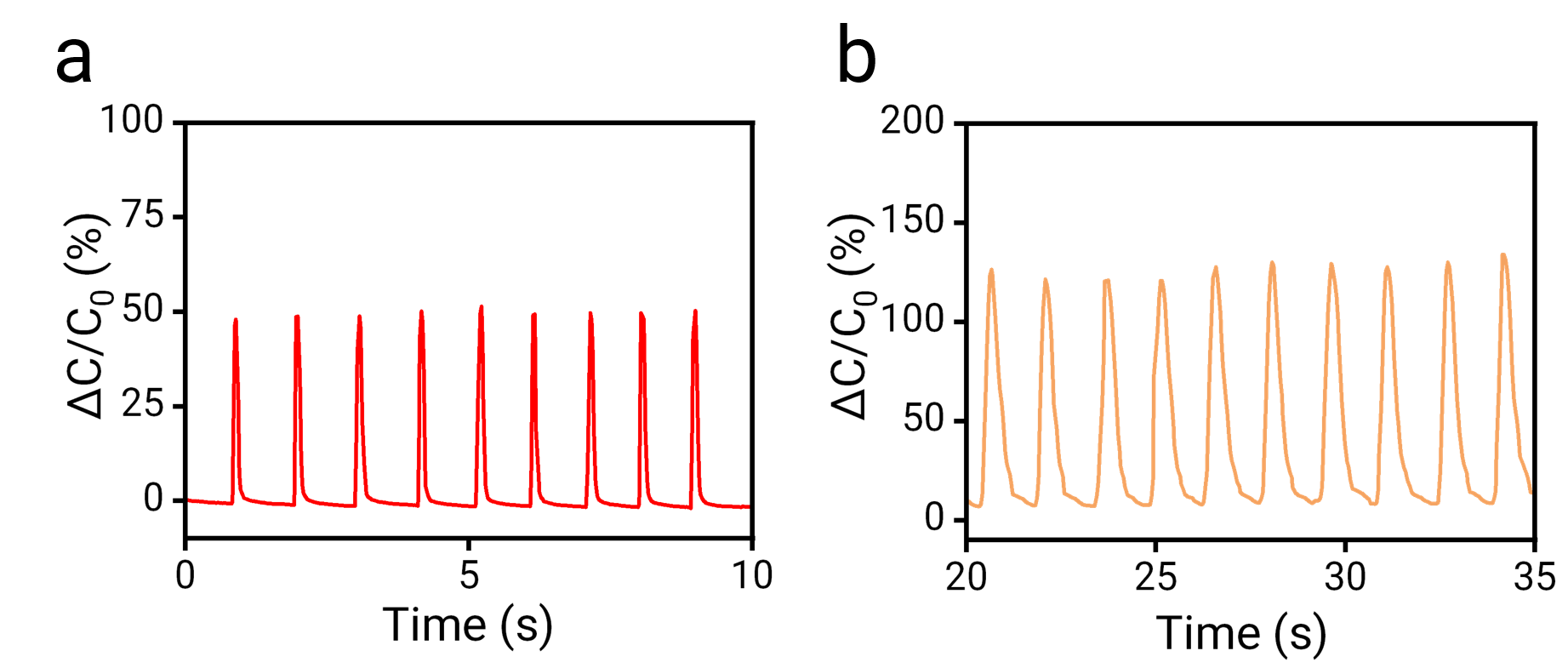


**Fig. S15 Capacitance response diagram of PGEH for human joint movement.** The recorded sensing response of the sensor for (**a**) knee bending and (**b**) wrist bending


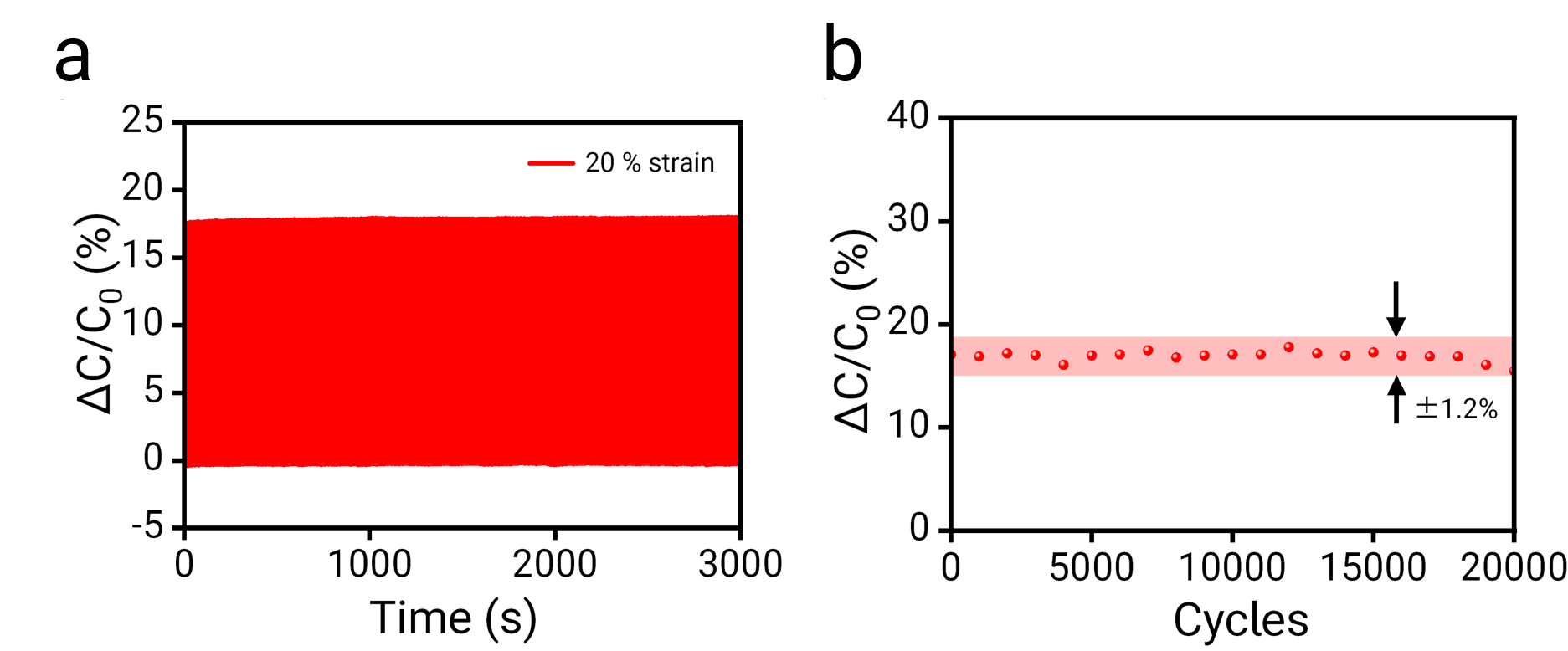


**Fig. S16 Long-term capacitance stability testing of PGEH sensor. a** Capacitance response of PGEH sensor with applied compressive strain ranging from 0 and 20% over a period of 3,000 s. **b** The capacitance variation range over 20,000 cycles is approximately 1.2%


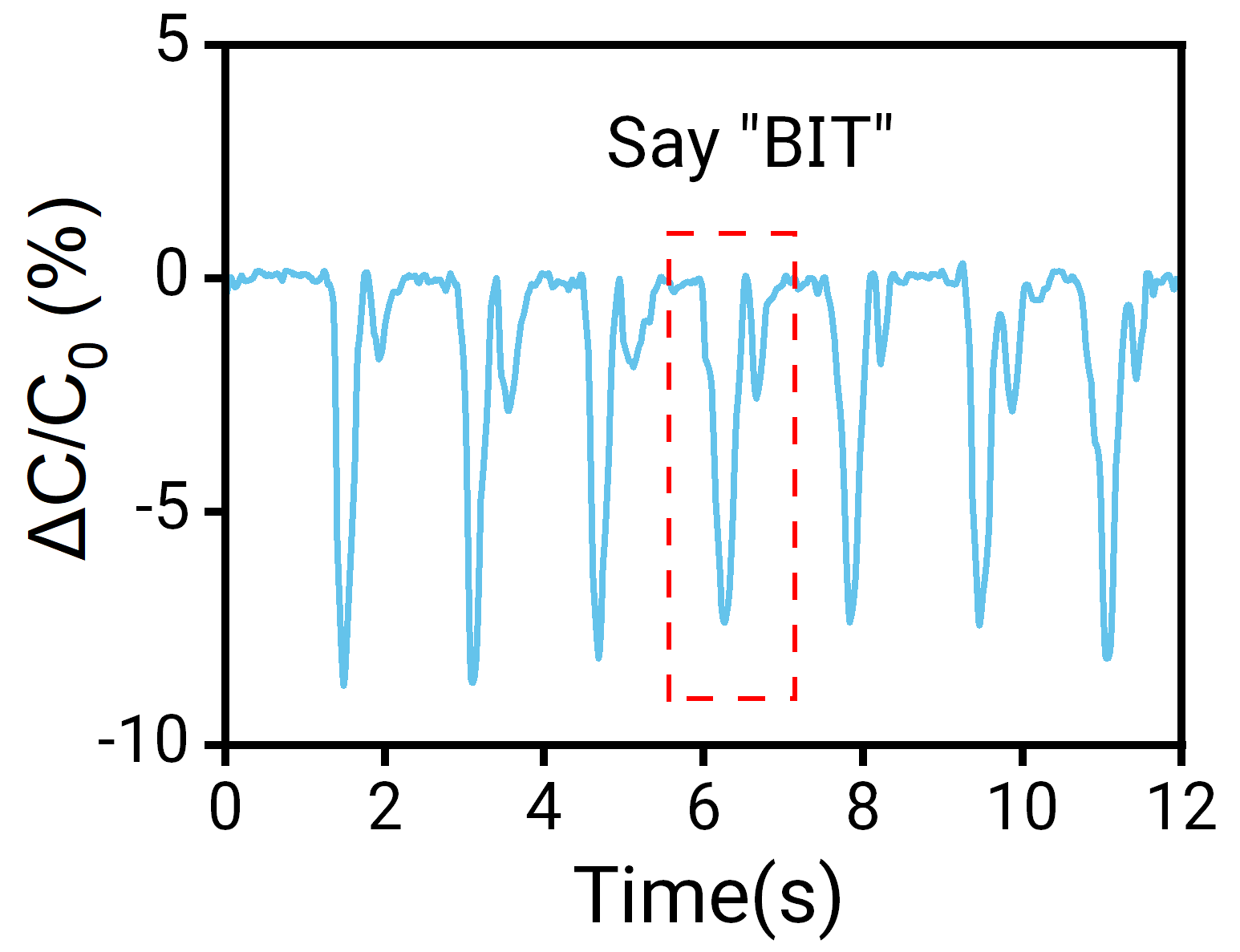


**Fig. S17 Capacitance response diagram for vocal cord vibrations.** PGEH adheres to the throat and responds to a continuous, stable waveform generated by vocal fold vibrations during articulation


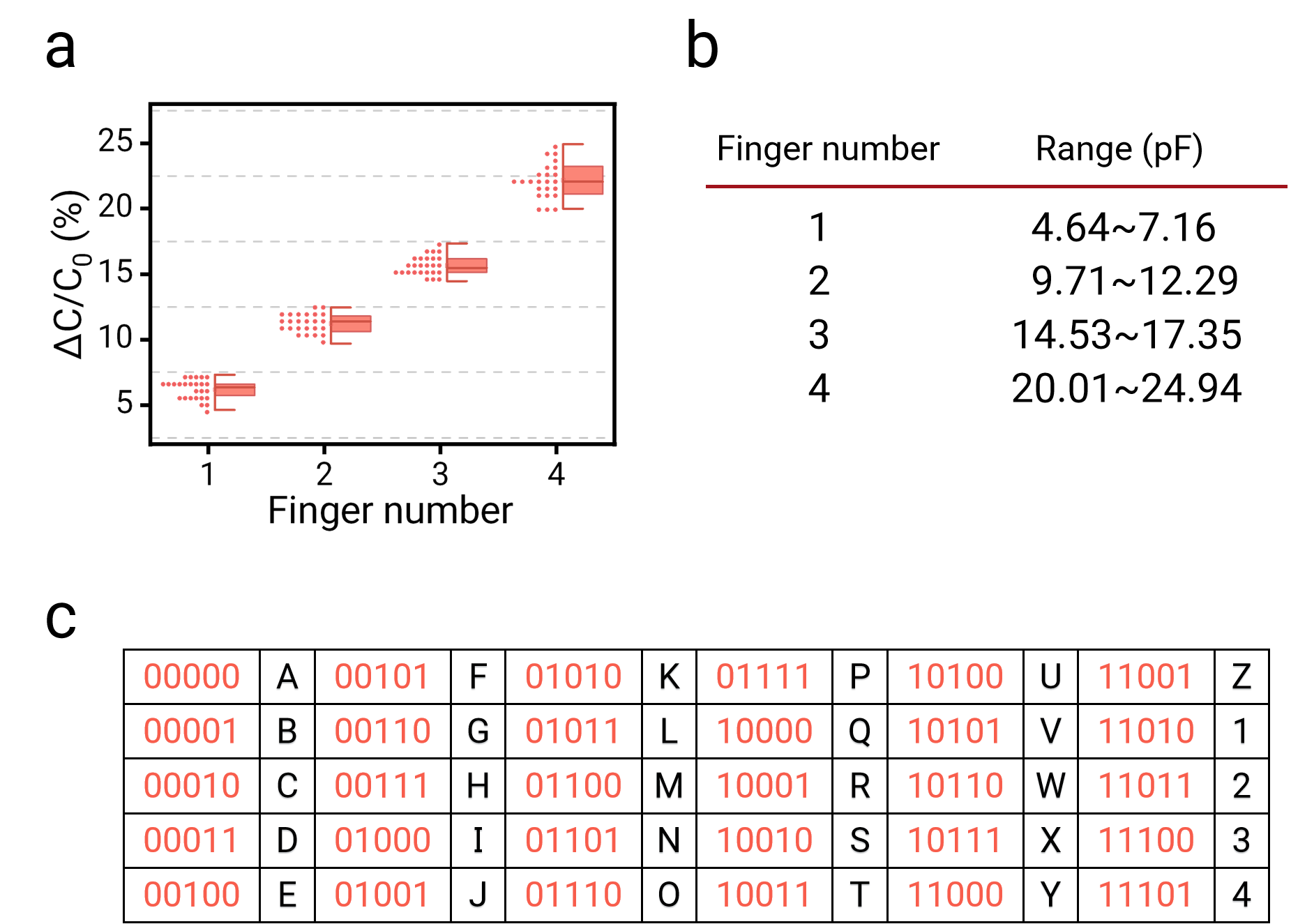


**Fig. S18 Box diagram of capacitance response under different number of fingers touches. a** Capacitance response range of PGEH sensor with different number of fingers. **b** Capacitance value response range. Data are reported as their means ± SDs (N ≥ 3). **c** Binary code comparison table

**Fig. S19 Intelligent coding system based on PGEH capacitive sensor. a** A image of touching the PGEH sensor with fingertips. **b** Schematic diagram of decoding and transmitting messages through PGEH sensor. **c, d** The schematic diagram of image transmitted using different codes: **c** Morse code, **d** binary code


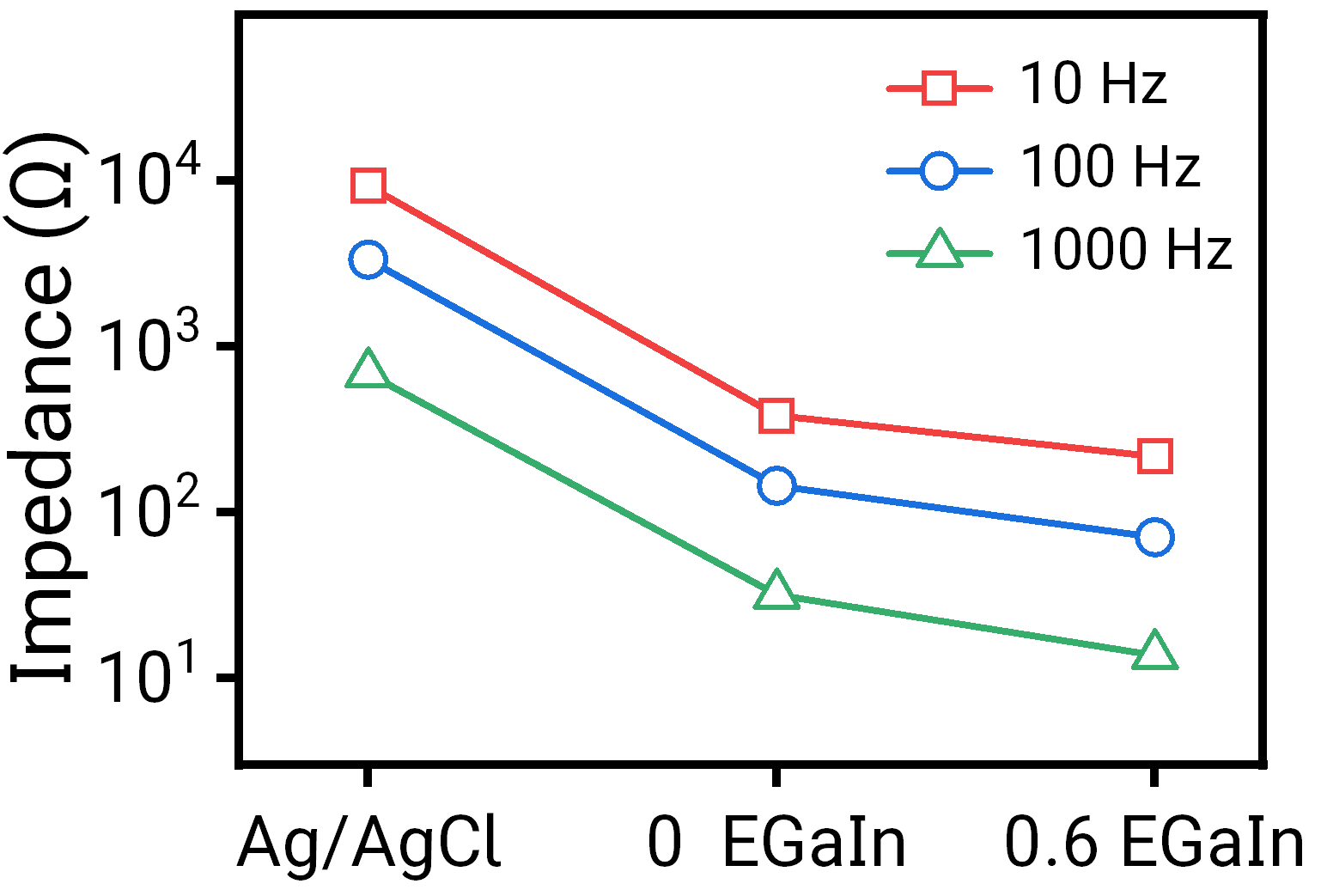


**Fig. S20 Comparison of electrical properties with commercial electrodes.** The impedance performance of Ag/AgCl, PGEH-0 EGaIn and PGEH-0.6 EGaIn was tested at 10, 100 Hz and 1,000 Hz


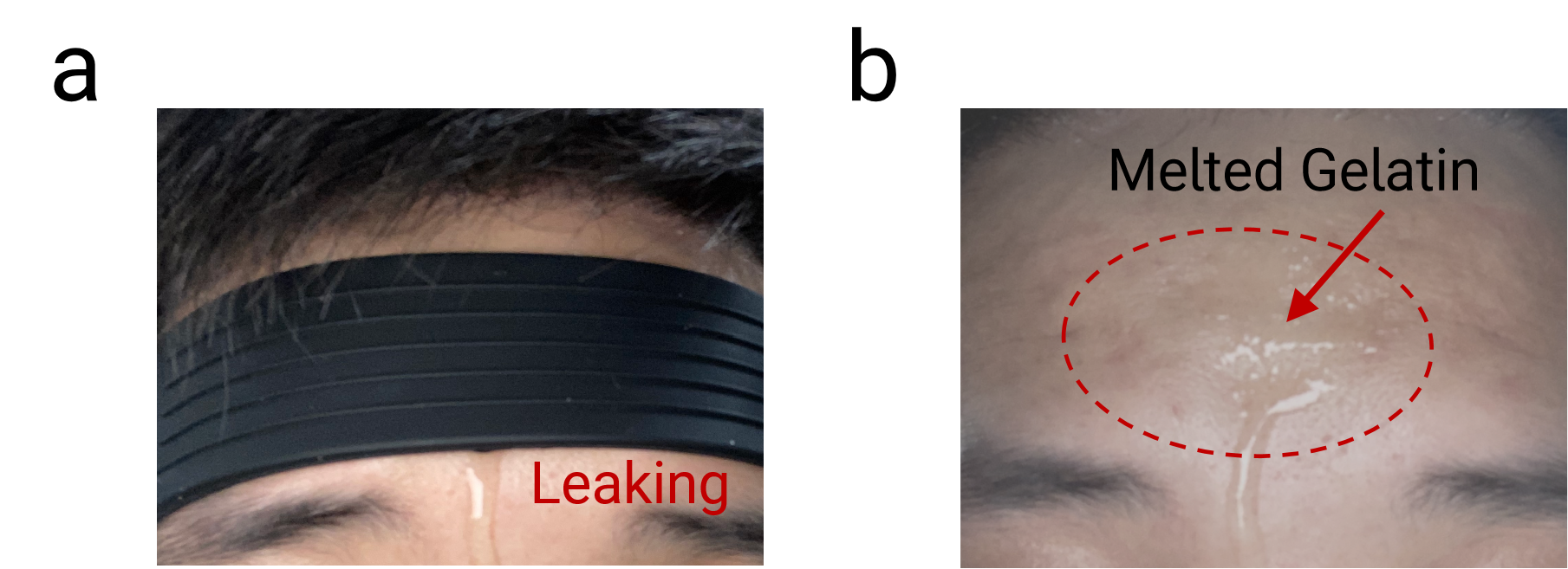


**Fig. S21 Photos of leakage from pure gelatin hydrogel during the EEG signal collection process.** **a** During the wearing process. **b** Removing the EEG acquisition device


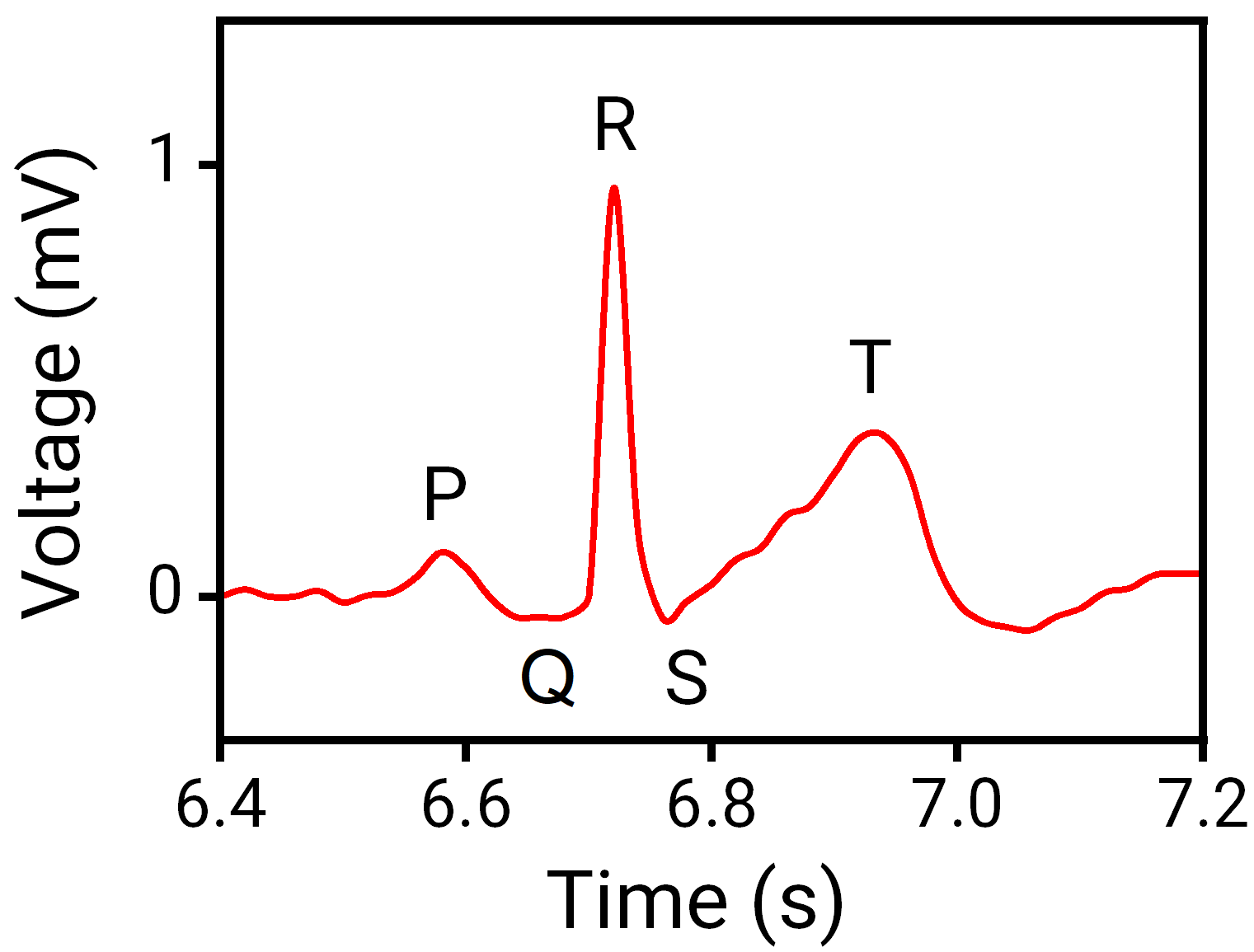


**Fig. S22 The wireless measured ECG signals.** The ECG signals acquired by PGEH, clearly presented P-wave, QRS complex, and T-wave

**
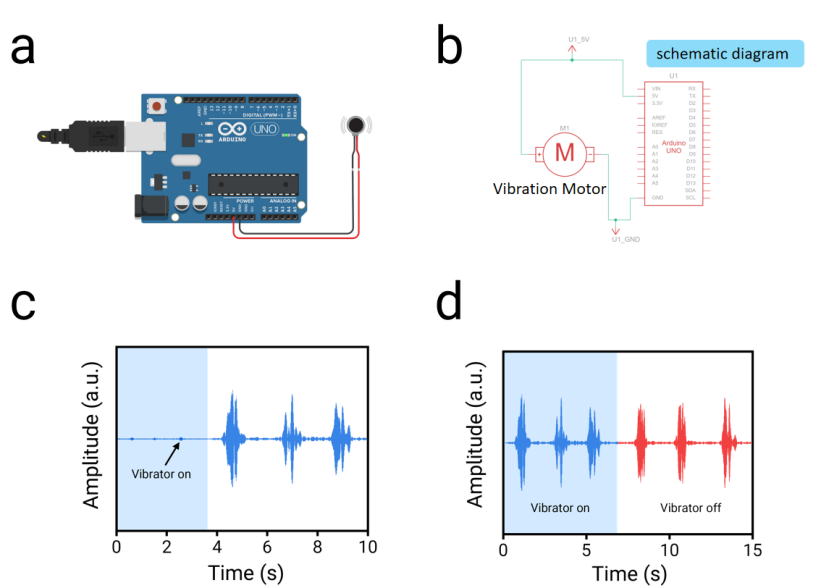
**

**Fig. S23 Vibration artifact interference assessment in EMG monitoring.** **a, b** Schematic and operational principle of the pulse vibration apparatus. **c** Comparative signal profiles from isolated vibration stimuli versus grip-force activity. **d** Synchronized EMG signal comparison during grip-force execution with/without concurrent vibration stimuli. We designed experimental validation using controlled vibration stimuli (Fig. S15a,b) to simulate subtle motion artifacts during EMG measurements. As shown in Fig. S15c, vibration-induced capacitive fluctuations produced signal artifacts with amplitudes comparable to baseline noise levels (<2% of EMG signal magnitude). Crucially, synchronized grip-force tests confirmed that these mechanical perturbations exerted no influence on EMG signal fidelity (Fig. S15d). These results substantiate that EMG signal acquisition remains robust against concurrent capacitive variations under dynamic monitoring scenarios

**
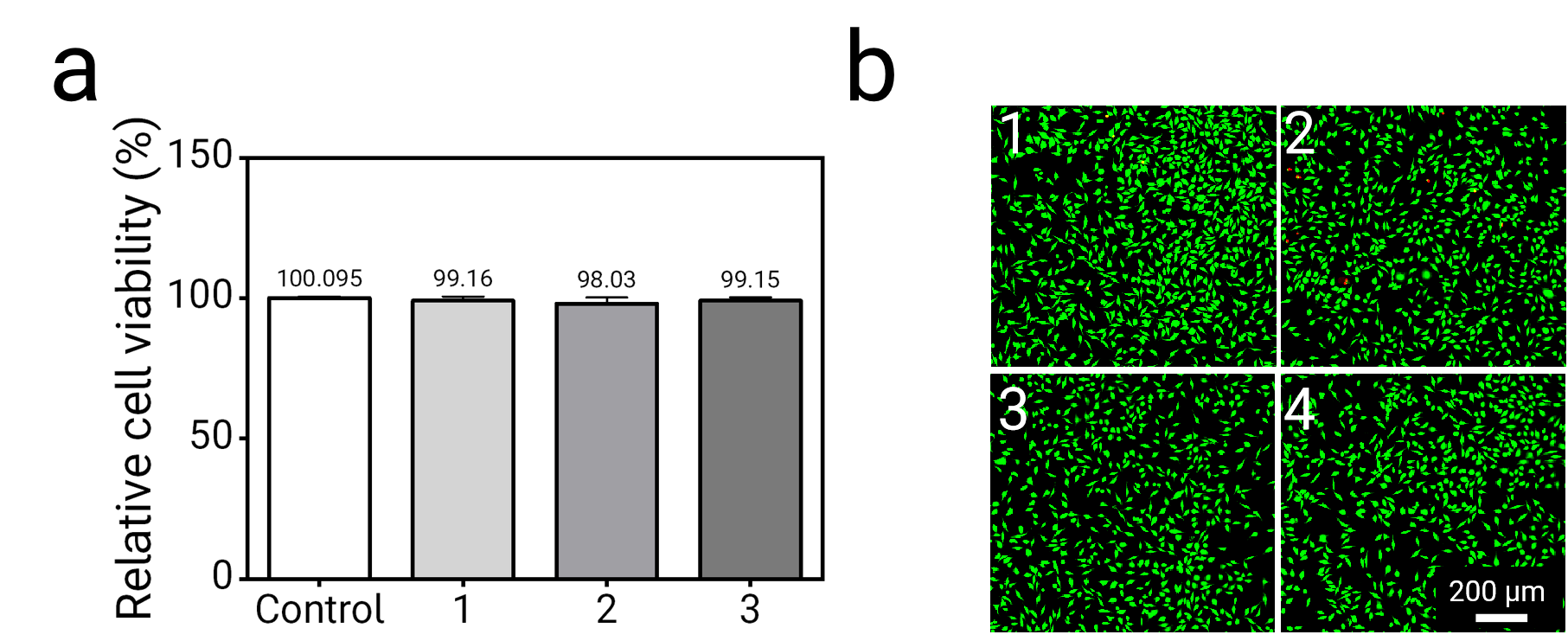
**

**Fig. S24 Cytotoxicity of the PGEH. a** Cell viability of leach liquors on L929 cells after culturing for 24 h was assessed using CCK-8 assays. The leach liquors were prepared by immersing 100 mg/mL hydrogels in phosphate buffer saline (PBS) at 37 ℃ for 24 hours. (1. Control group, 2. PAM, 3. Gelatin 4. PGEH). Data are presented as mean values ± S.D. (n = 3). **b** Live/dead staining confocal images (live: green; dead: red) of L929 cells after incubation with leach liquors for 24 h. Cytotoxicity testing was performed according to the ISO 10993-5 international standard with leach liquor from the hydrogels. The experiment results revealed that minimal dead cells were detectable after incubation with the leach liquor of the hydrogels for 24 hours, thus demonstrating that the hydrogels did not induce detectable cell cytotoxicity.


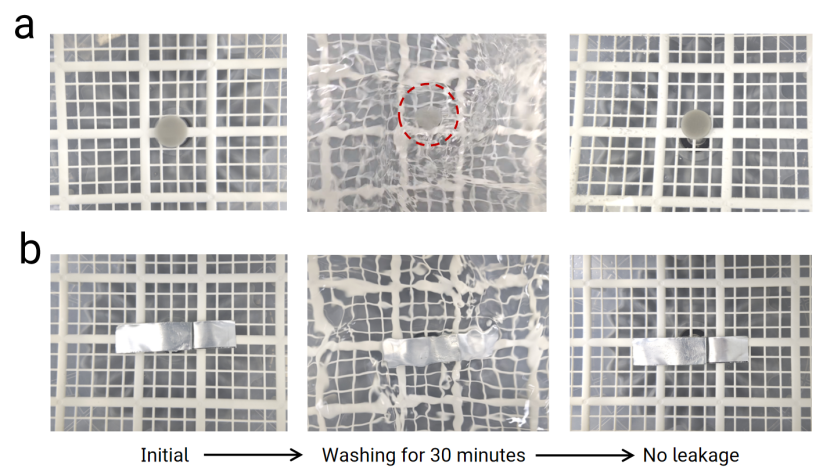


**Fig. S25 Leakage resistance evaluation of PGEH hydrogel. a** Hydrogel samples and corresponding assembled sensor. **b** were immersed in a water-filled container and subjected to continuous water flow for 30 minutes to examine potential leakage of liquid metal components.


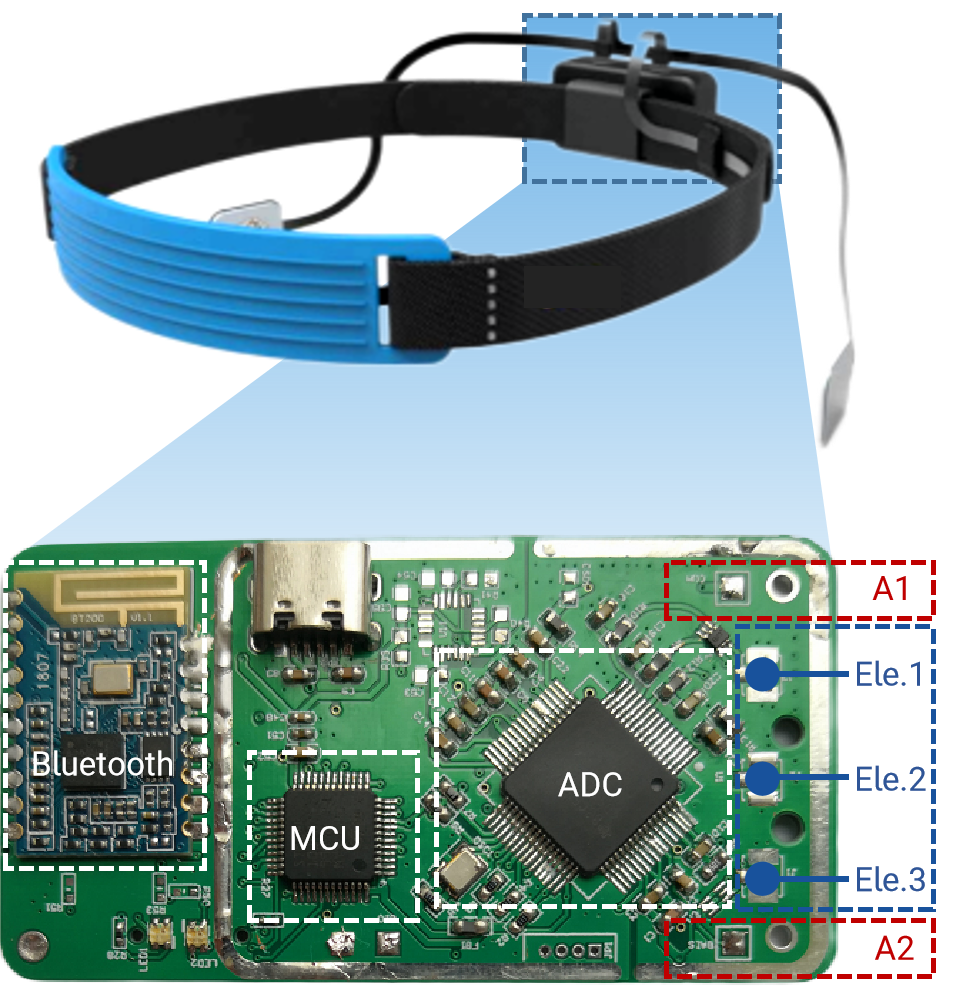


**Fig. S26 Hardware composition of EEG signal acquisition headband.** (Top) Photographs of EEG signal acquisition headband. (Bottom) Hardware composition diagram of EEG acquisition headband with three-channel interface


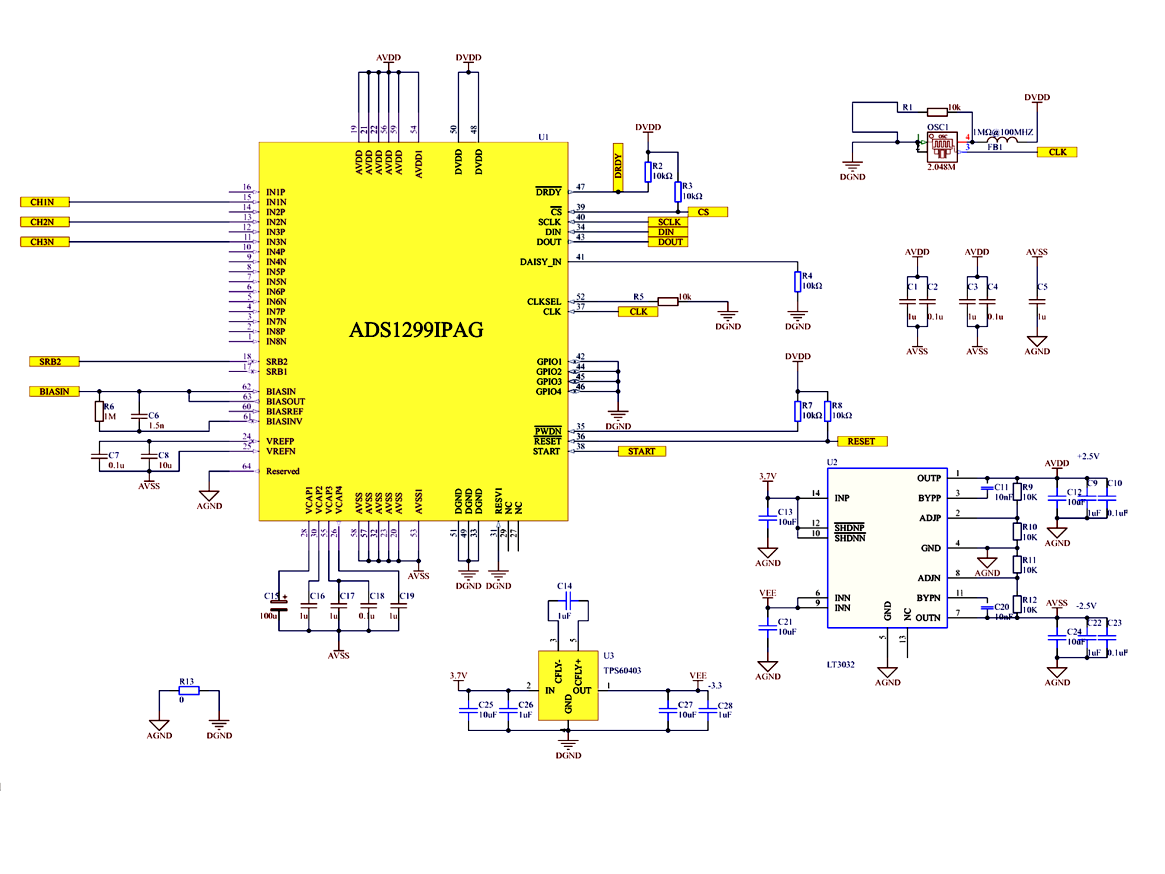


**Fig. S27 Circuit diagram of the EEG signal analysis system.** To acquire low-back noise and high signal-to-noise ratio (SNR) EEG data, the three-lead EEG sensor employs the ADS1299-8PAG (manufactured by Texas Instruments, Inc.) as the analog-to-digital converter (ADC) for EEG signal digitization. The sensor utilizes the LT3032 (manufactured by analog devices, Inc.) to provide a ±2.5 V power supply for the ADS1299-8PAG. To balance performance and power consumption, the EEG sensor incorporates a 32-bit ARM Cortex MCU (STM32F103C8T6, manufactured by STMicroelectronics, Inc.) to manage peripheral components, including the ADC and Bluetooth module.


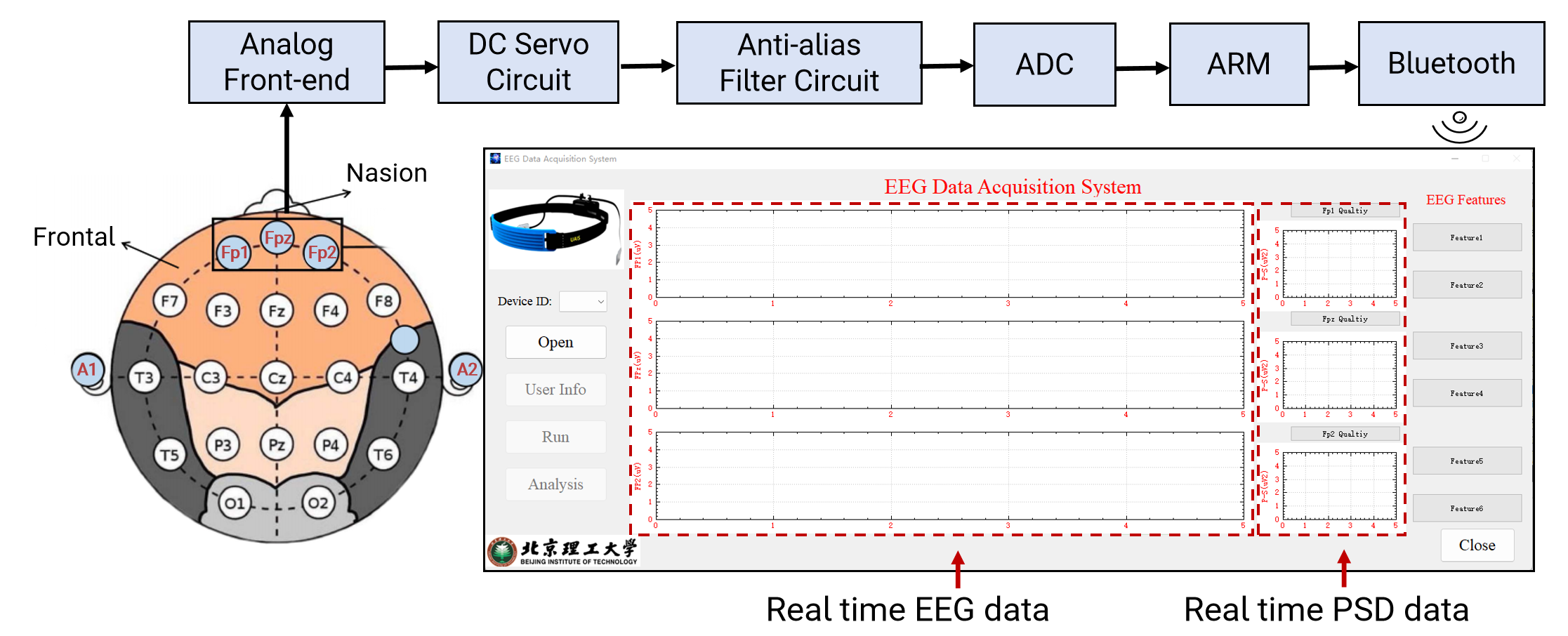


**Fig. S28 Primary hardware components of the EEG sensor and schematic diagram of Graphic user interface.** EEG signals are first processed by the analog front-end (AFE) for impedance matching and direct current offset reduction, then amplified to fit the dynamic range of ADC. They are filtered to prevent aliasing and digitized by ADC. The data is sent to a host computer via Bluetooth. A 32-bit Arm Cortex MCU (micro control unit) manages peripherals to for balance performance and power consumption. The sensor runs on a 600 mAh lithium polymer battery, regulated by Linear and low-dropout (LDO) regulators for different components. All parts are assembled on two 55×28mm^2^ PCBs (printed circuit boards). Three PGEH electrode are mounted at the Fp2, Fpz, and Fp1 sites for EEG recording. The other two working and reference electrodes are placed behind two ears to effectively eliminate hair interference. High-quality EEG signals collected by the headband were used to establish the dataset for attention assessment.


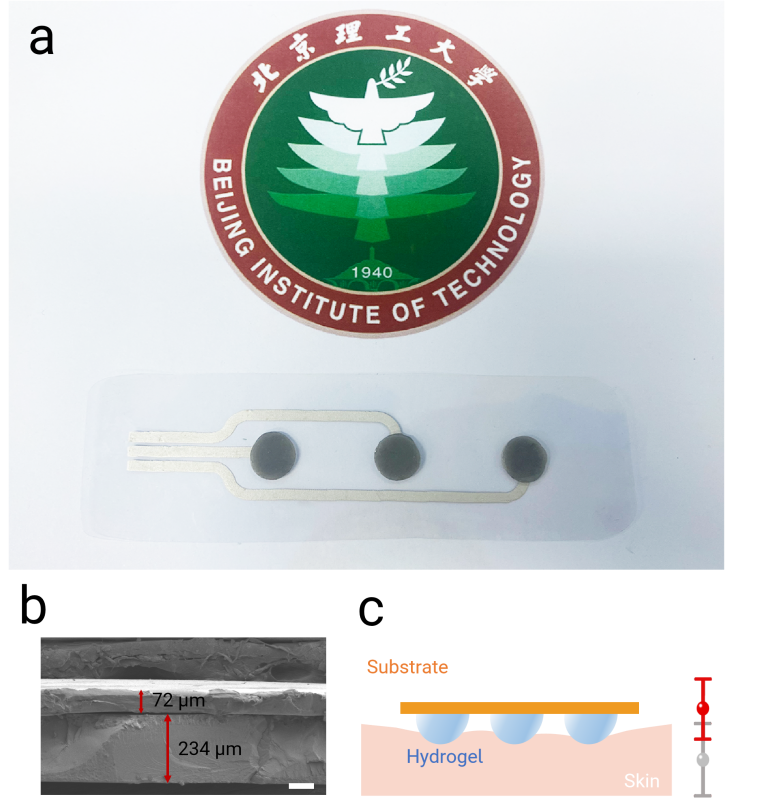


**Fig. S29 Photographs of ultrathin PGEH and schematic diagram of skin interface of PGEH electrodes.** The PEGH electrode can be formed by dropping hydrogel onto the surface of the PU printed electrode, creating an ultra-thin PEGH electrode that significantly reduces gel interface impedance. **a** Optical image of PGEH electrode. **b** Microscope image of the PGEH electrode. **c** Schematic diagram of PGEH electrode skin contact.


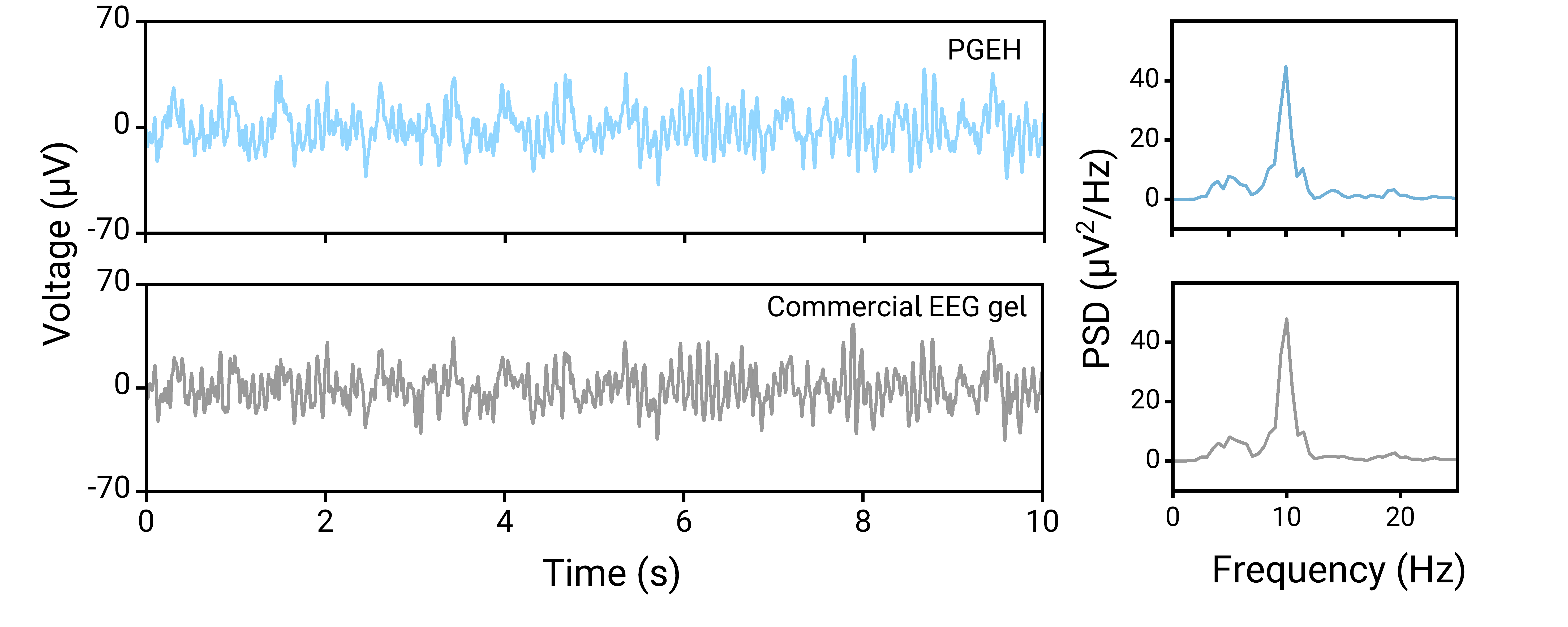


**Fig. S30 EEG recording by PGEH and commercial electrodes.** EEG alpha rhythms recorded using the PGEH (Top) and commercial EEG gel (Bottom). Power spectral density (PSD) is calculated from EEG signals recorded by electrodes painted with PGEH (top) and commercial EEG gel (bottom). Distinct alpha rhythm features, at approximately 10 Hz, are observable in EEG signals during closed-eyes conditions


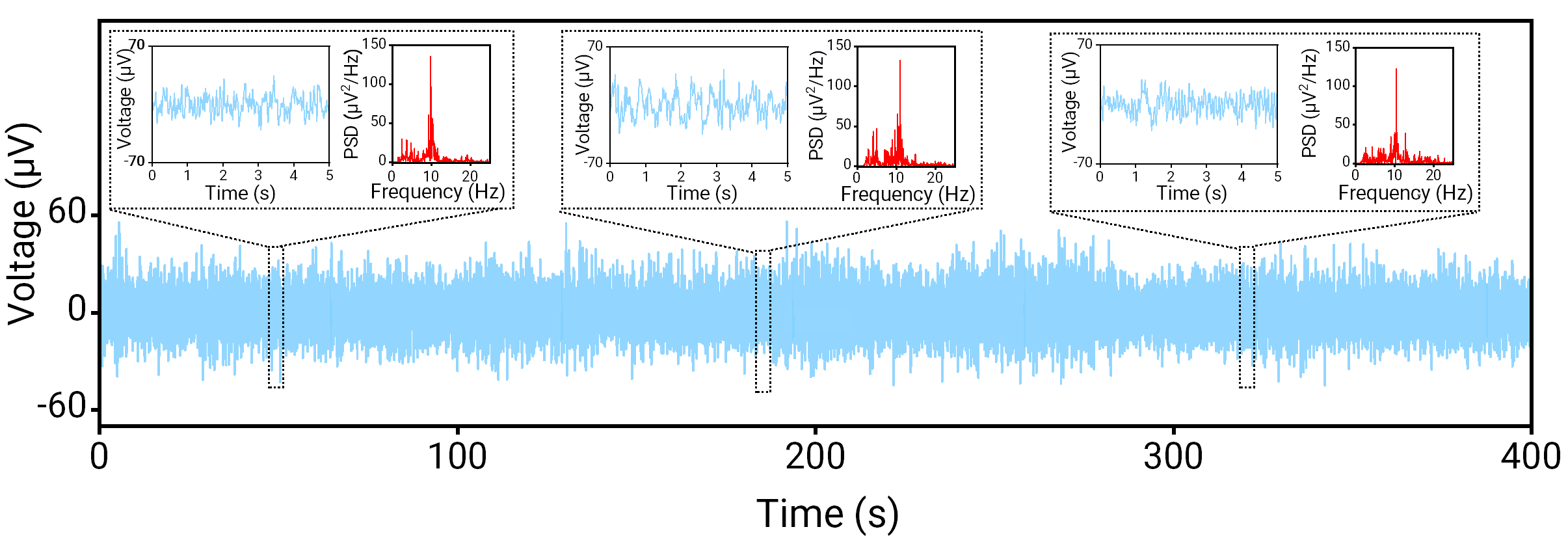


**Fig. S31 Long-period EEG alpha rhythms continuously recorded by PGEH electrode for 0, 24 and 48 hours.** The PGEH electrode showed superior recording stability and the insets show the zoomed-in data segments and their corresponding PSD


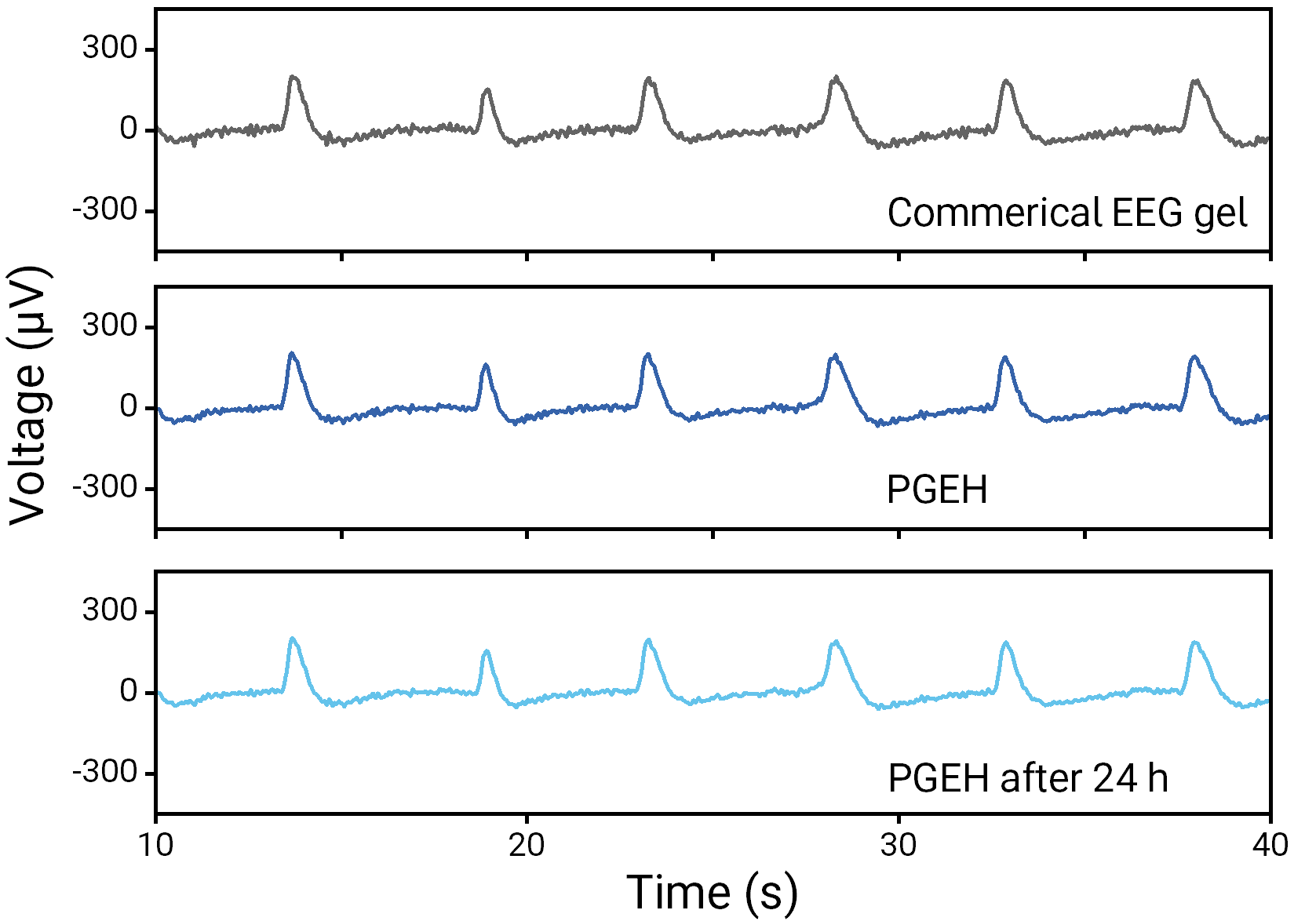


**Fig. S32 Electrooculogram (EOG) recording by PGEH and commercial electrodes.** The representative periodic EOG signals captured by the PGEH and commercial gels are readily observable during eye blink movements


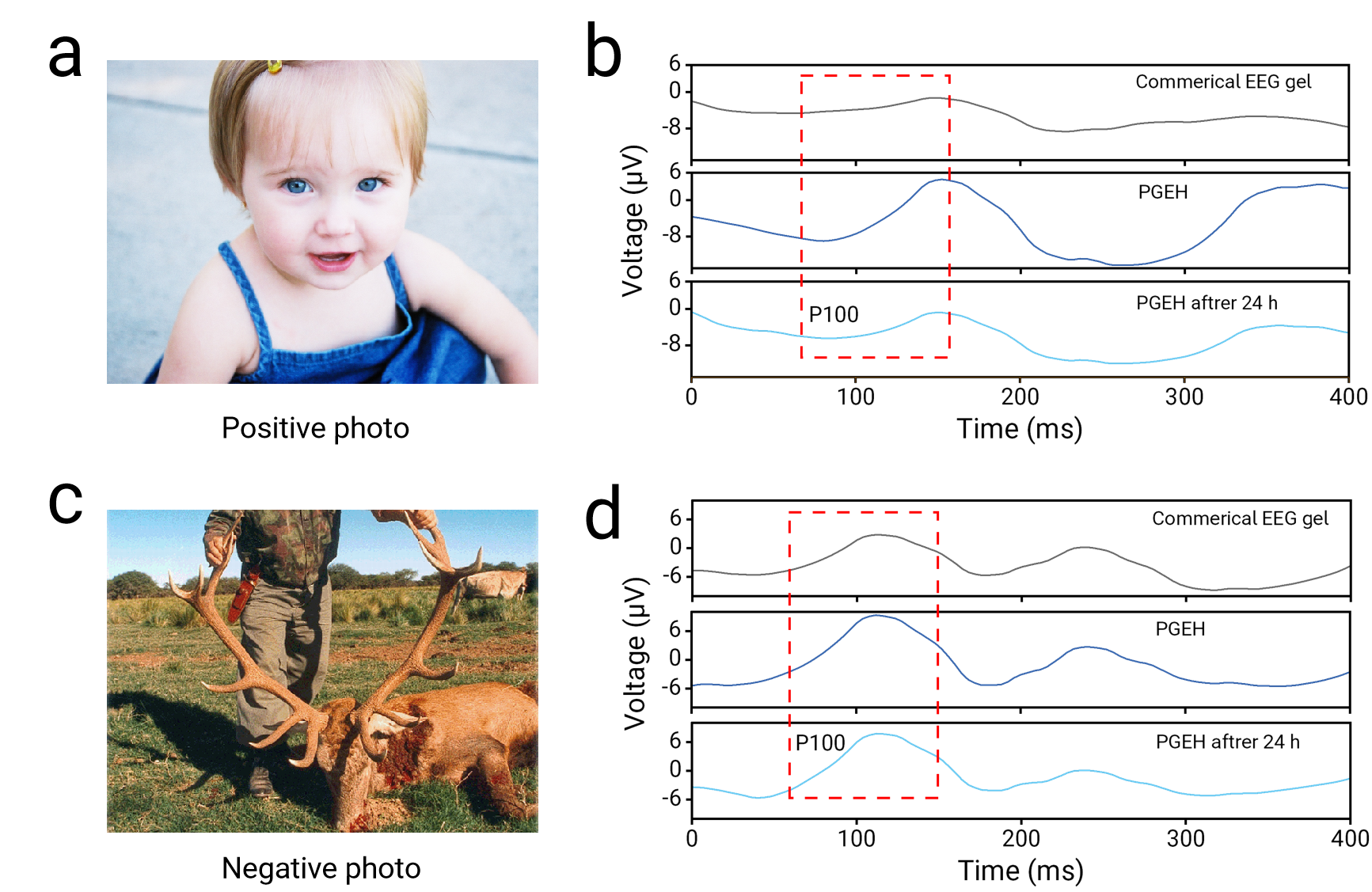


**Fig. S33 Visual evoked potential (VEP) signals recording by PGEH and commercial electrodes.** **a** Positive photos and negative photos **c** of the experimental setup for measuring P100 signals. **b** and **d** P100 signals recorded by commercial EEG gel and PGEH. All mentioned physiological markers were monitored throughout 24 hours of continuous wearing. a.u., arbitrary units. When observing delightful or sad photos, the characteristic P100 waveforms appeared for both PGEH and commercial gels within the normal range of 66 to 124 ms. (P100 represents the transient potential of VEP with a latency of about 100 ms)


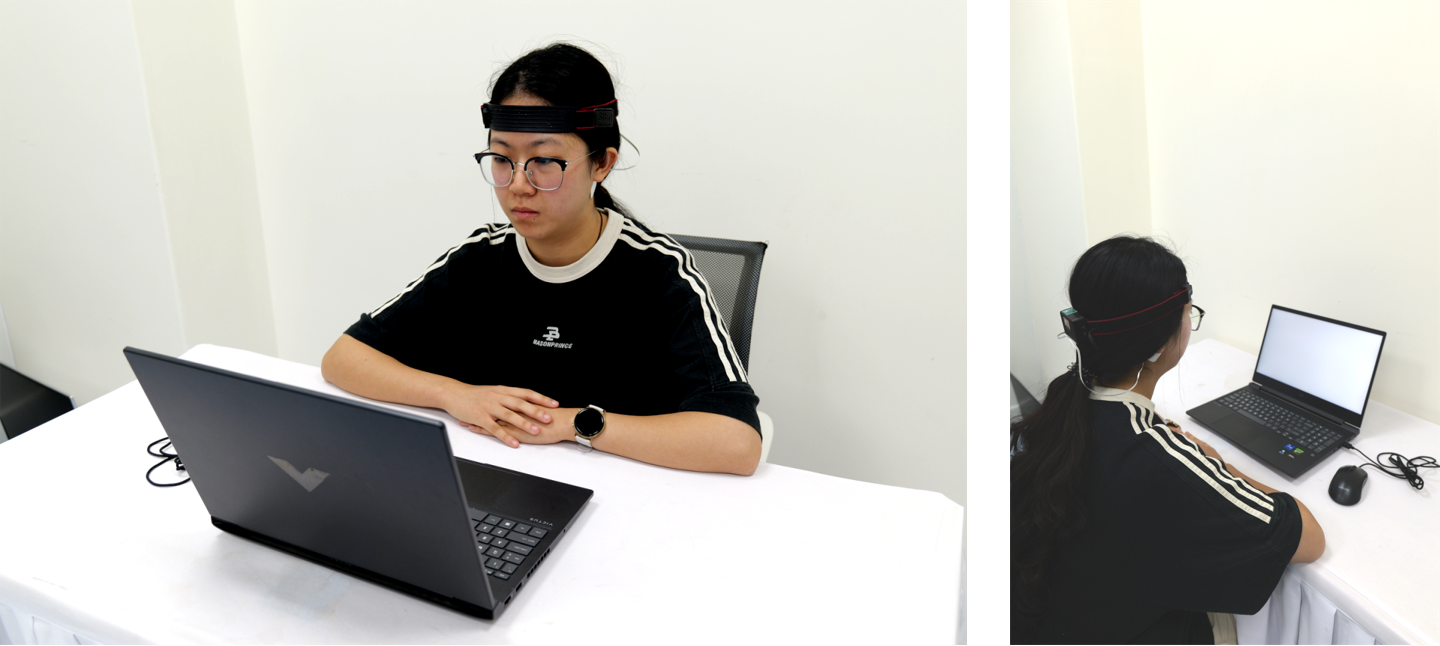


**Fig. S34 Environmental photographs of participants engaged in attention assessment paradigm.** During the experiment, the participants were instructed to remain seated in a relaxed but attentive manner, with the EEG helmet securely fitted. They were positioned approximately 60 centimeters away from the monitor, which displayed the stimuli in a controlled environment to minimize external distractions. Their focus was directed towards a blank screen to ensure minimal interference during data collection


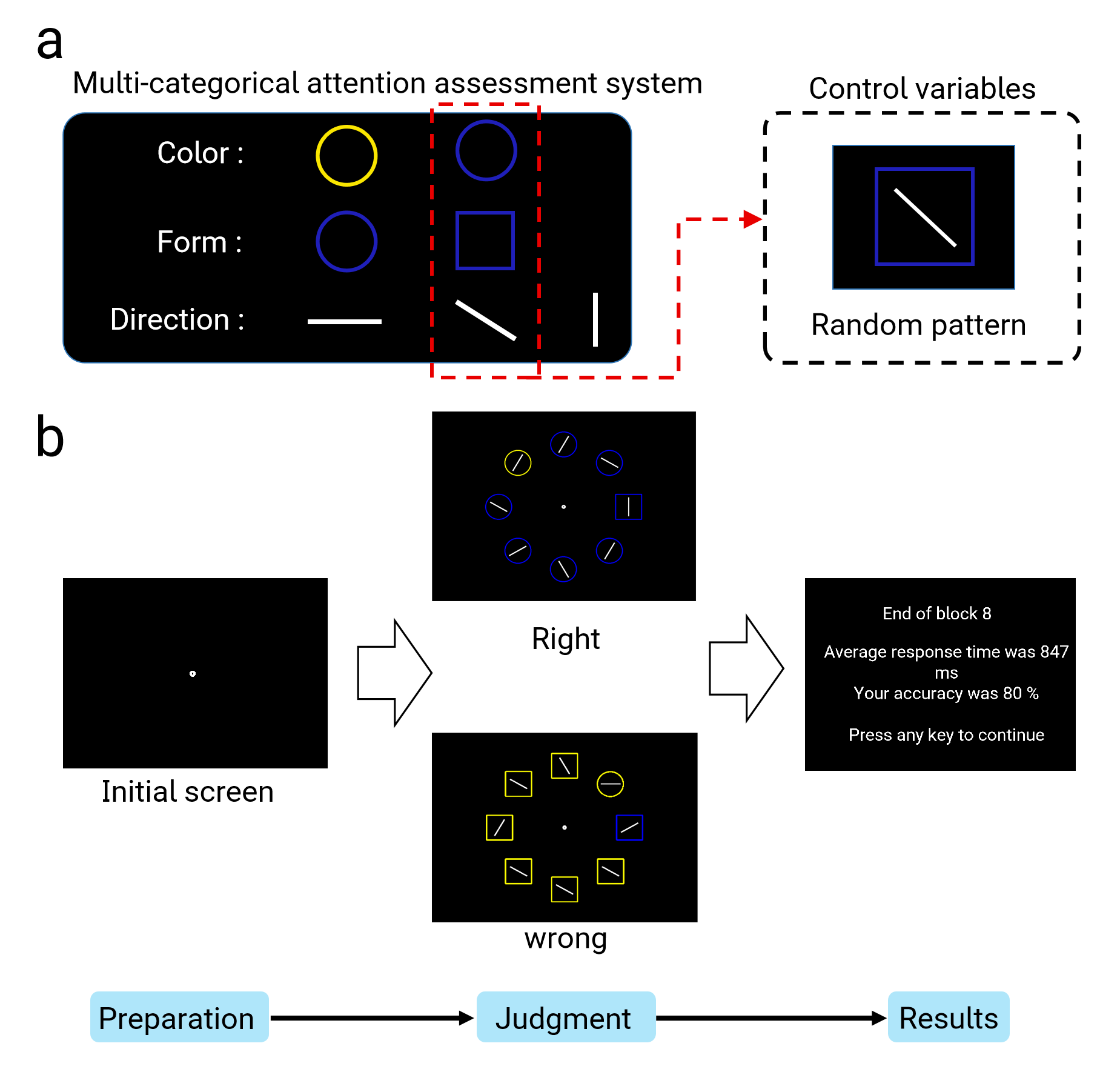


**Fig. S35 Schematic diagram illustrating the principle of attention assessment paradigm. a** Paradigm demands that participants focus fully on observing the screen, selecting the correct image from randomly generated patterns featuring various colors, shapes, and angles. **b** Photographs of the attention assessment paradigm show 10 groups, each consisting of 10 cycles, with the accuracy of each group being recorded


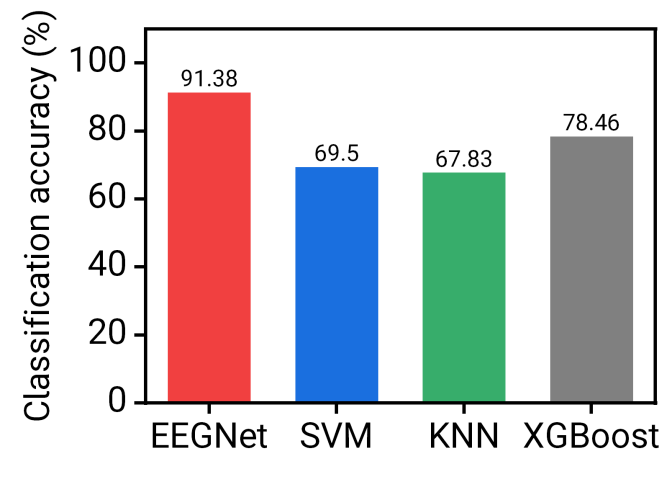


**Fig. S36 Recognition accuracy statistics of different algorithms.** We employed several machine learning models to evaluate discrimination accuracy, specifically EEGNet, support vector machine (SVM), extreme gradient boosting (XGBoost), and K-nearest neighbors (KNN). The recognition accuracies obtained were as follows: 91.38% for EEGNet, 69.5% for SVM, 67.83% for KNN, and 78.46% for XGBoost

1. EEGNet: EEGNet is a deep neural network specifically designed for EEG signal classification. It consists of convolutional layers that capture both temporal and spatial features of EEG signals. The network uses lightweight structures and optimized layers to improve computational efficiency and accuracy, particularly in scenarios with small datasets.

2. Support Vector Machine (SVM): SVM is a supervised learning model used for classification tasks. It works by finding the optimal hyperplane that separates data points of different classes in a high-dimensional space. SVM is known for its effectiveness in high-dimensional spaces and its ability to handle non-linear relationships through kernel functions.

3. K-Nearest Neighbors (KNN): KNN is a simple, instance-based learning algorithm that classifies a data point based on the majority class of its KNN in the feature space. It is easy to implement and interpret but can become computationally expensive as the dataset size grows.

4. Extreme Gradient Boosting (XGBoost): XGBoost is a powerful machine learning model based on gradient boosting. It combines multiple weak classifiers to form a strong predictive model by sequentially correcting the errors made by previous classifiers. XGBoost is known for its high performance and speed, especially when dealing with large datasets and complex features.


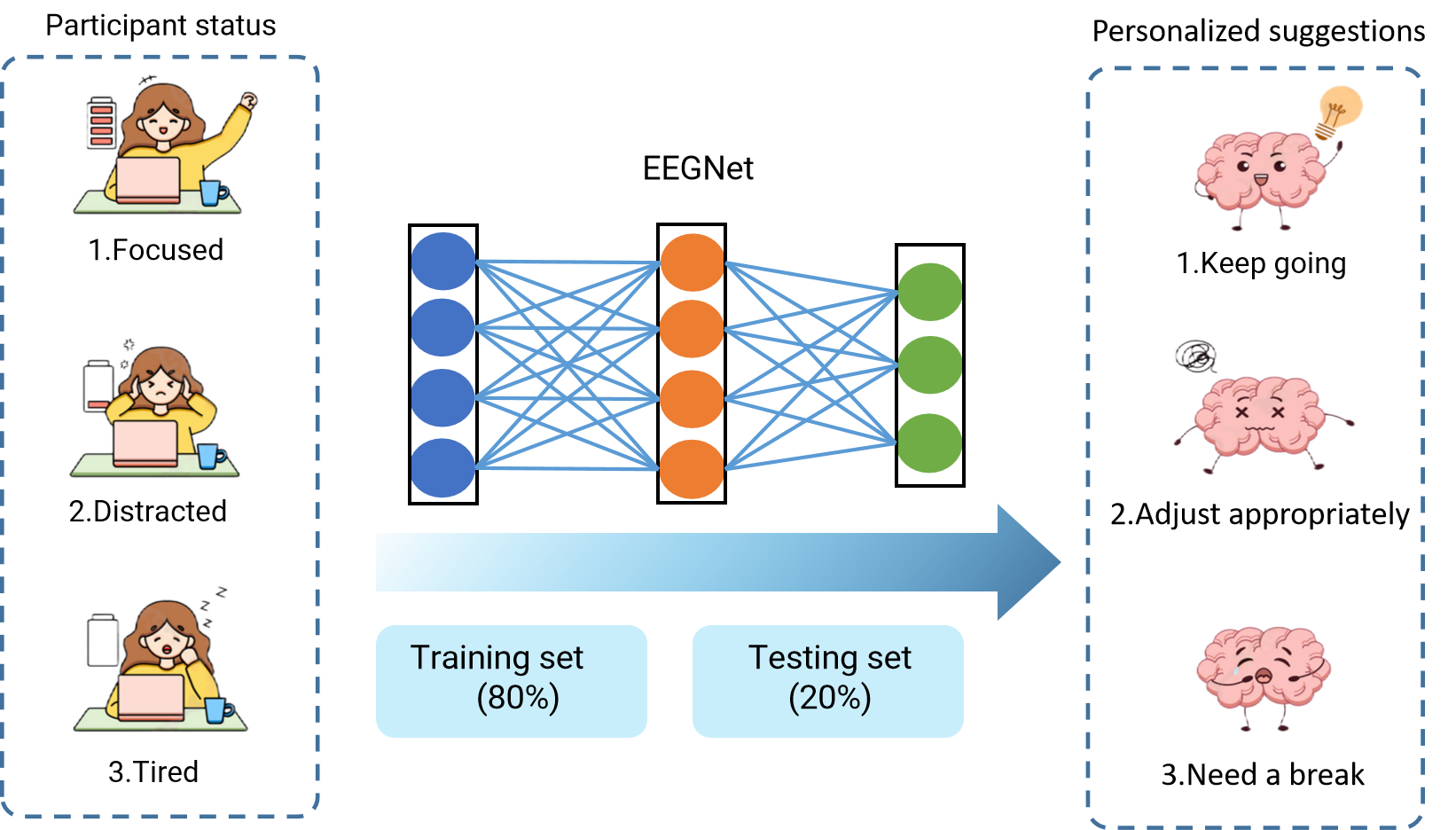


**Fig. S37 Schematic diagram of personalized attention regulation feedback utilizing the EEGNet neural network.** By leveraging the analytical capabilities of the EEG Net machine learning algorithm, we can provide more individualized assessments of attentional focus. These customized evaluations are crucial for delivering precise analytical insights and targeted support to individuals with diverse needs

**Table S1** Interaction energies of different complexes

| Model | Distance (Å) | Energy (kcal/mol) |
| --- | --- | --- |
| PAM-Gly | 1.588 | -26.21 |
| PAM-Hyp | 1.953 | 24.77 |
| PAm-Pro | 1.652 | -18.53 |

**Table S2 Comparisons of representative adhesive hydrogels**

| Materials | Elongation at break (%) | Stress (KPa) | Adhesion strength (KPa) | Reusable adhesion  (cycle) | Thermo-sensitive  adhesion |
| --- | --- | --- | --- | --- | --- |
| MXene/HA-PBA/TA [S6] | 240 | 27 | 10.17 | 10 | N/A |
| PDEH-SLM [S7] | 1760 | 71.2 | 29.2 | 10 | N/A |
| PR-Gel [S8] | 830 | 78.1 | 4.5 | N/A | N/A |
| PAM/DAC [S8] | 520 | 47 | ~8 | N/A | N/A |
| PVA/SA/TA/Ca [S9] | ~450 | 210.61 | 75.08 | N/A | N/A |
| PEDOT: PSS/PDA [S10] | 800 | ~10 | 46.45 ± 1.75 | 300 | Yes |
| PAM/Gelatin [S11] | 1424 | 129.1 | ~52 | N/A | Yes |
| SC [S12] | 1800 | 47 | ~30 | 50 | Yes |
| **This work** | **1643** | **366.54** | **104** | **30** | **Yes** |

Note: ‘N/A’ indicates ‘not available’ in the references.

**Table S3** Comparisons of representative pressure sensors

| Materials | Type of pressure sensor | Bio compatibility | Maximum sensibility (KPa^-1^) | Response (ms) | Stability |
| --- | --- | --- | --- | --- | --- |
| PI-PMF [S13] | Resistive | N/A | 2.02e-4 | 100 | 10,000 cycles |
| CIP/NdFeB/PD [S14] | Capacitive | N/A | 0.314 | 200 | 5,000 cycles |
| GelMA [S15] | Capacitive | N/A | 0.19 | 161 | 3,000 cycles |
| SBS/Ag/PD [S16] | Capacitive | N/A | 0.21 | 40 | 10,000 cycles |
| LM/PVA [S17] | Capacitive | N/A | 1.24 | 100 | 10,000 cycles |
| PVDF@AgNWs@TiO [S18] | Capacitive | N/A | 0.0012 | 167 | 30,000 cycles |
| CCNFs [S19] | Resistive | N/A | 0.053 | 375 | 1,000 cycles |
| MRDN [S20] | Resistive | N/A | 0.925 | 750 | 600 cycles |
| PSeD-U [S21] | Capacitive | Yes | 0.16 | 100 | - |
| **This work** | **Capacitive** | **Yes** | **1.25** | **30** | **20,000 cycles** |

Note: ‘N/A’ indicates ‘not available’ in the references.

**Table S4** Comparisons of representative epidermal electrodes

| Materials | Application | Bio compatibility | Skin interfacial impedance | Signal quality | Long-term  durability |
| --- | --- | --- | --- | --- | --- |
| MXene/HA-PBA/TA [S6] | EEG/ECG/EMG | Yes | At 100 Hz: ~11 kΩ, 100 kΩ ^(a)^ | 35.63 dB, 4.75 dB ^(a)^ | N/A |
| PDEH-SLM [S7] | EMG | Yes | At 1 kHz: ~9.1 kΩ, ~30.3 kΩ ^(a)^ | 25.52 dB, 22.12 dB ^(a)^ | 4 days |
| AHBH-ECC [S22] | EEG/EMG | Yes | At 1 kHz: 4.8 kΩ, 12.0 kΩ ^(a)^ | ~9.5 μV^2^, ~46.3 μV^2^ ^(a)^ | 7 days |
| PEDOT: PSS/ WPU/  D-sorbitol [S23] | ECG/EMG/  EEG | N/A | At 10 Hz:82 kΩ cm^2^, 148 kΩ cm^2 (a)^ | <38 μV^2^ | N/A |
| Au/Parylene [S24] | ECG/  EMG | Yes | At 1 kHz: ~44 kΩ, >100 kΩ ^(a)^ | 8.77–8.79 μV^2^, 7.7–37.8 μV^2^ ^(a)^ | N/A |
| Ag/PBAc-SA [S25] | EMG | Yes | At 1 Hz: ~4 kΩ, ~8 kΩ ^(a)^ | N/A | N/A |
| PVA/PVP/PDA NPs [S18] | EEG | Yes | At 100 Hz:4 kΩ, 8 kΩ ^(a)^ | 2.85 μV^2^, 2000.47 μV^2^ ^(a)^ | 14 day |
| pDAM/Au/PDMS [S26] | ECG | Yes | At 1 kHz: ~20 kΩ, 10 kΩ ^(a)^ | N/A | 15 min (under water) |
| **This work** | **EEG/ECG/EMG** | **Yes** | **At 100 Hz:71 Ω, 36 kΩ ^(a)^** | **25.82 dB, 21.05 dB ^(a)^**  **7.3 μV^2^ , 55.7 μV^2^ ^(a)^** | **14 day** |

(a) Commercial gel Ag/AgCl electrodes or standard wet Ag/AgCl electrodes

Note: ‘N/A’ indicates ‘not available’ in the references

**Supplementary References**

1. K.K. Parhi, M. Ayinala, Low-complexity welch power spectral density computation. IEEE Trans. Circuits Syst. I Regul. Pap. **61**(1), 172–182 (2014). <https://doi.org/10.1109/TCSI.2013.2264711>
2. J. Theeuwes, Perceptual selectivity for color and form. Percept. Psychophys. **51**(6), 599–606 (1992). <https://doi.org/10.3758/BF03211656>
3. U. Korn, M. Krylova, K.L. Heck, F.B. Häußinger, R.S. Stark et al., EEG-microstates reflect auditory distraction after attentive audiovisual perception recruitment of cognitive control networks. Front. Syst. Neurosci. **15**, 751226 (2021). <https://doi.org/10.3389/fnsys.2021.751226>
4. M.A. Francisco-Vicencio, F. Góngora-Rivera, X. Ortiz-Jiménez, D. Martinez-Peon, Sustained attention variation monitoring through EEG effective connectivity. Biomed. Signal Process. Control **76**, 103650 (2022). <https://doi.org/10.1016/j.bspc.2022.103650>
5. V.J. Lawhern, A.J. Solon, N.R. Waytowich, S.M. Gordon, C.P. Hung et al., EEGNet: a compact convolutional neural network for EEG-based brain–computer interfaces. J. Neural Eng. **15**(5), 056013 (2018). <https://doi.org/10.1088/1741-2552/aace8c>
6. Y. Zhang, Z. Li, Z. Xu, M. Xiao, Y. Yuan et al., Flexible healable electromagnetic-interference-shielding bioelastic hydrogel nanocomposite for machine learning-assisted highly sensitive sensing bioelectrode. Aggregate **5**(5), e566 (2024). <https://doi.org/10.1002/agt2.566>
7. G. Yang, Z. Lan, H. Gong, J. Wen, B. Pang et al., A *Nepenthes*-inspired hydrogel hybrid system for sweat-wicking electrophysiological signal recording during exercises. Adv. Funct. Mater. **35**(13), 2417841 (2025). <https://doi.org/10.1002/adfm.202417841>
8. H. Shi, H. Huo, H. Yang, H. Li, J. Shen et al., Cellulose-based dual-network conductive hydrogel with exceptional adhesion. Adv. Funct. Mater. **34**(48), 2408560 (2024). <https://doi.org/10.1002/adfm.202408560>
9. R. Song, X. Wang, M. Johnson, C. Milne, A. Lesniak-Podsiadlo et al., Enhanced strength for double network hydrogel adhesive through cohesion-adhesion balance. Adv. Funct. Mater. **34**(23), 2313322 (2024). <https://doi.org/10.1002/adfm.202313322>
10. X. Xiong, Y. Chen, Z. Wang, H. Liu, M. Le et al., Polymerizable rotaxane hydrogels for three-dimensional printing fabrication of wearable sensors. Nat. Commun. **14**(1), 1331 (2023). <https://doi.org/10.1038/s41467-023-36920-3>
11. Y. Zhang, Y. Dai, F. Xia, X. Zhang, Gelatin/polyacrylamide ionic conductive hydrogel with skin temperature-triggered adhesion for human motion sensing and body heat harvesting. Nano Energy **104**, 107977 (2022). <https://doi.org/10.1016/j.nanoen.2022.107977>
12. L. Bai, Y. Jin, X. Shang, H. Jin, L. Shi et al., Temperature-triggered smart milk-derived hydrogel with programmable adhesion for versatile skin-attached iontronics. Nano Energy **104**, 107962 (2022). <https://doi.org/10.1016/j.nanoen.2022.107962>
13. M. Cai, Z. Jiao, S. Nie, C. Wang, J. Zou et al., A multifunctional electronic skin based on patterned metal films for tactile sensing with a broad linear response range. Sci. Adv. **7**(52), eabl8313 (2021). <https://doi.org/10.1126/sciadv.abl8313>
14. B. Ji, Q. Zhou, B. Hu, J. Zhong, J. Zhou et al., Bio-inspired hybrid dielectric for capacitive and triboelectric tactile sensors with high sensitivity and ultrawide linearity range. Adv. Mater. **33**(27), 2100859 (2021). <https://doi.org/10.1002/adma.202100859>
15. Z. Li, S. Zhang, Y. Chen, H. Ling, L. Zhao et al., Gelatin methacryloyl-based tactile sensors for medical wearables. Adv. Funct. Mater. **30**(49), 2003601 (2020). <https://doi.org/10.1002/adfm.202003601>
16. Q. Liang, D. Zhang, Y. Wu, X. Qu, Y. Jia et al., Stretchable helical fibers with skin-core structure for pressure and proximity sensing. Nano Energy **113**, 108598 (2023). <https://doi.org/10.1016/j.nanoen.2023.108598>
17. K. Zheng, F. Gu, H. Wei, L. Zhang, X.-A. Chen et al., Flexible, permeable, and recyclable liquid-metal-based transient circuit enables contact/noncontact sensing for wearable human-machine interaction. Small Methods **7**(4), e2201534 (2023). <https://doi.org/10.1002/smtd.202201534>
18. F. Wang, Y. Xue, X. Chen, P. Zhang, L. Shan et al., 3D printed implantable hydrogel bioelectronics for electrophysiological monitoring and electrical modulation (adv. funct. mater. 21/2024). Adv. Funct. Mater. **34**(21), 2470116 (2024). <https://doi.org/10.1002/adfm.202470116>
19. Q. Li, B. Tian, G. Tang, H. Zhan, J. Liang et al., Multifunctional conductive hydrogels for wearable sensors and supercapacitors. J. Mater. Chem. A **12**(6), 3589–3600 (2024). <https://doi.org/10.1039/d3ta06771h>
20. J. Zheng, G. Chen, H. Yang, C. Zhu, S. Li et al., 3D printed microstructured ultra-sensitive pressure sensors based on microgel-reinforced double network hydrogels for biomechanical applications. Mater. Horiz. **10**(10), 4232–4242 (2023). <https://doi.org/10.1039/d3mh00718a>
21. S. Chen, L. Sun, X. Zhou, Y. Guo, J. Song et al., Mechanically and biologically skin-like elastomers for bio-integrated electronics. Nat. Commun. **11**(1), 1107 (2020). <https://doi.org/10.1038/s41467-020-14446-2>
22. G. Yang, K. Zhu, W. Guo, D. Wu, X. Quan et al., Adhesive and hydrophobic bilayer hydrogel enabled on-skin biosensors for high-fidelity classification of human emotion. Adv. Funct. Mater. **32**(29), 2200457 (2022). <https://doi.org/10.1002/adfm.202200457>
23. H. Yang, S. Ji, I. Chaturvedi, H. Xia, T. Wang et al., Adhesive biocomposite electrodes on sweaty skin for long-term continuous electrophysiological monitoring. ACS Mater. Lett. **2**(5), 478–484 (2020). <https://doi.org/10.1021/acsmaterialslett.0c00085>
24. R.A. Nawrocki, H. Jin, S. Lee, T. Yokota, M. Sekino et al., Self-adhesive and ultra-conformable, sub-300 nm dry thin-film electrodes for surface monitoring of biopotentials. Adv. Funct. Mater. **28**(36), 1803279 (2018). <https://doi.org/10.1002/adfm.201803279>
25. Y. Xue, J. Zhang, X. Chen, J. Zhang, G. Chen et al., Trigger-detachable hydrogel adhesives for bioelectronic interfaces. Adv. Funct. Mater. **31**(47), 2106446 (2021). <https://doi.org/10.1002/adfm.202106446>
26. S. Ji, C. Wan, T. Wang, Q. Li, G. Chen et al., Water-resistant conformal hybrid electrodes for aquatic endurable electrocardiographic monitoring. Adv. Mater. **32**(26), e2001496 (2020). <https://doi.org/10.1002/adma.202001496>
